# PssJ Is a Terminal Galactosyltransferase Involved in the Assembly of the Exopolysaccharide Subunit in *Rhizobium leguminosarum* bv. *Trifolii*

**DOI:** 10.3390/ijms21207764

**Published:** 2020-10-20

**Authors:** Małgorzata Marczak, Magdalena Wójcik, Kamil Żebracki, Anna Turska-Szewczuk, Kamila Talarek, Dominika Nowak, Leszek Wawiórka, Marcin Sieńczyk, Agnieszka Łupicka-Słowik, Kamila Bobrek, Marceli Romańczuk, Piotr Koper, Andrzej Mazur

**Affiliations:** 1 Department of Genetics and Microbiology, Institute of Biological Sciences, Maria Curie-Skłodowska University, Akademicka 19 St., 20-033 Lublin, Poland; magdalena.wojcik@poczta.umcs.lublin.pl (M.W.); kamilzebracki@poczta.umcs.lublin.pl (K.Ż.); aturska@hektor.umcs.lublin.pl (A.T.-S.); kamilatalarek@gmail.com (K.T.); domi.com@wp.pl (D.N.); marcel.romanczuk11@gmail.com (M.R.); piotr.koper@poczta.umcs.lublin.pl (P.K.); mazur@hektor.umcs.lublin.pl (A.M.); 2 Department of Molecular Biology, Institute of Biological Sciences, Maria Curie-Skłodowska University, Akademicka 19 St., 20-033 Lublin, Poland; mniak11@hektor.umcs.lublin.pl; 3 Department of Organic and Medical Chemistry, Faculty of Chemistry, Wrocław University of Science and Technology, Norwida 4/6 St., 50-373 Wrocław, Poland; marcin.sienczyk@pwr.edu.pl (M.S.).; agnieszka.lupicka-slowik@pwr.edu.pl (A.Ł.-S.); 4 Department of Epizootiology and Clinic of Bird and Exotic Animals, Faculty of Veterinary Medicine, Wroclaw University of Environmental and Life Sciences, Norwida 31 St., 50-375 Wrocław, Poland; kamila.bobrek@upwr.edu.pl

**Keywords:** exopolysaccharide, glycosyltransferase, *Rhizobium*, symbiosis

## Abstract

*Rhizobium leguminosarum* bv. *trifolii* produces exopolysaccharide (EPS) composed of glucose, glucuronic acid, and galactose residues at a molar ratio 5:2:1. A majority of genes involved in the synthesis, modification, and export of exopolysaccharide are located in the chromosomal Pss-I region. In the present study, a Δ*pssJ* deletion mutant was constructed and shown to produce EPS lacking terminal galactose in the side chain of the octasaccharide subunit. The lack of galactose did not block EPS subunit translocation and polymerization. The *in trans* delivery of the *pssJ* gene restored the production of galactose-containing exopolysaccharide. The mutant was compromised in several physiological traits, e.g., motility and biofilm production. An impact of the *pssJ* mutation and changed EPS structure on the symbiotic performance was observed as improper signaling at the stage of molecular recognition, leading to formation of a significant number of non-infected empty nodules. Terminal galactosyltransferase PssJ was shown to display a structure typical for the GT-A class of glycosyltransferases and interact with other GTs and Wzx/Wzy system proteins. The latter, together with PssJ presence in soluble and membrane protein fractions indicated that the protein plays its role at the inner membrane interface and as a component of a larger complex.

## 1. Introduction

Exopolysaccharides (EPSs) are extracellular carbohydrate polymers produced, secreted, and accumulated outside the cells of various microorganisms. The huge structural diversity among EPS is related to the monosaccharide composition and linkage in the single subunit, the repeating unit size, the degree of polymerization, and non-carbohydrate decorations [1]. A variety of structural combinations mediates plenty of EPS properties and functions, including protection against environmental stresses (e.g., osmotic stress, temperature, pH, and damage by UV light, heavy metals, oxidants, or desiccation), cell adherence to surfaces and to each other, formation of a biofilm matrix, storage of carbon and water, and nutrient uptake [2,3,4,5].

EPS is of special importance for soil-dwelling and nitrogen-fixing bacteria, which are able to establish symbiotic interactions with legume plants, collectively called rhizobia. In addition to its numerous beneficial properties, which are helpful for rhizobia during their free-living life stage in a challenging soil environment, exopolysaccharide constitutes the key determinant of successful interactions between symbiotic partners [6,7,8].

The basic octasaccharide subunit of acidic EPS produced by the clover symbiont *Rhizobium leguminosarum* bv. *trifolii* is composed of D-glucose, D-glucuronic acid, and D-galactose residues in a molar ratio 5:2:1 and additionally modified with O-acetyl and pyruvyl groups [9,10,11,12]. To date, the crucial steps towards EPS subunit biosynthesis in *R. leguminosarum* have been only partially recognized. A majority of genes involved in the synthesis, modification, and export of EPS are located in the large chromosomal Pss-I cluster, which is well conserved among various *R. leguminosarum* and *R. etli* strains [13,14,15]. Pss-I contains an almost entire set of genes encoding putative glycosyltransferases (GTs) responsible for EPS subunit synthesis. GTs represent a diverse class of enzymes that catalyze the synthesis of glycosidic linkages by the transfer of a sugar residue from a donor substrate to an acceptor. They play essential roles in the biosynthesis of oligo- and polysaccharides, protein glycosylation, and formation of valuable natural products [16]. According to the Carbohydrate-Active enZYmes database (CAZy) [17], the currently recognized GTs are classified into 111 families. In contrast to glycosylhydrolases (also carbohydrate-active enzymes) that adopt a variety of folds (all α, all β, or mixed α/β structures), GT folds have been observed to consist primarily of α/β/α sandwiches, similar or very close to the Rossmann-type fold, i.e., a classical structural motif of a six-stranded parallel β-sheet with 321456 topology found in many nucleotide-binding proteins [18]. The structures of all GTs resolved to date adopt one of three folds termed GT-A, GT-B, and GT-C [16,19]. GT-A and GT-B differ substantially in the number and organization of β/α/β Rossmann-like domains forming their active centers and their divalent metal ion dependency. The limited number of structures among GTs may result from the evolutionary origin of only few precursor sequences [20]. Despite the low identity of nucleotide and amino acid sequences, even if they synthesize the same glycan linkages, similarities between GTs can be identified at a structural level [19,21]. Referring to the anomeric configuration of the new glycosidic bond synthesized in relation to the linkage in the donor, a distinction between “inverting” and “retaining” GTs have been made [22]. Noteworthy, inverting and retaining enzymes are present in both the GT-A and GT-B families, indicating that the different domain organization does not correlate with the catalytic mechanism [16].

It has been assumed that GTs responsible for the biosynthesis of EPS in *R. leguminosarum* form a large multienzymatic complex located in the bacterial inner membrane [23]. The synthesis of EPS repeating units is initiated by priming glucosyl-IP-transferase PssA (encoded beyond the Pss-I cluster), which transfers glucose to the lipid anchor undPP [24,25,26]. Glucuronosyl-β-1,4-glucosyltransferase PssDE transfers glucuronic acid to glucose [26,27,28]. It is worth mentioning that PssD and PssE share sequence similarities with respective N- and C-terminal domains of known glucosyltransferases and the functionality of the PssDE complex has been confirmed by reciprocal genetic complementation of a mutation in a gene coding for glucuronosyl-β-1,4-glucosyltransferase SpsK involved in the sphingan synthesis in *Sphingomonas* S88 [26]. The transfer of another glucuronic acid residue is catalyzed by glucuronosyl-β-1,4-glucuronosyltransferase PssC [26]. Most likely, glucosyltransferase PssS is responsible for addition of the last glucose residue to the main chain of the EPS subunit [29]. Mutations in *pssA*, *pssE*, *pssD*, and *pssS* (but not *pssC*) totally abolish EPS synthesis [24,27,28,29]. Sequence analyses allowed implying that the subsequent steps of the assembly of the subunit side chain could be carried out by the PssF, PssG, PssH, PssI, and PssJ proteins sharing sequence similarity with GTs [23]. As a result of the similarity of PssJ with putative galactosyltransferases, it was nominated as candidate for the enzyme participating in the last step of the unit assembly [23]. Previously, a transposon mutant of *R. leguminosarum* bv. *trifolii* RBL5515 strain producing EPS repeating units lacking terminal galactose in the side chain was described [9]; however, direct evidence demonstrating engagement of putative galactosyltransferase encoded by *pssJ* in this process was not provided. Likewise, the information concerning interactions between glycosyltransferases within the postulated multiprotein complex and proteins active in the later stages of the subunit synthesis is still fragmentary [30]. For example, from among the PssR, PssM, and PssK proteins, most probably engaged in non-sugar modification of EPS, only PssM has been experimentally proved to serve the ketal pyruvate transferase function and modify the subterminal sugar residue in the repeating unit [31].

Similarly to other representatives of *Rhizobiaceae*, *R. leguminosarum* strains synthesize EPS in high-molecular-weight (HMW) and low-molecular-weight (LMW) forms, differing in the subunit polymerization degree [32,33]. Proteins constituting the EPS polymerization and export machinery, i.e., PssL flippase [34], polysaccharide polymerase PssT [35], co-polymerase PssP [32], and outer membrane channel protein PssN [36] are also encoded within the Pss-I gene cluster. In contrast to the poorly recognized stage of EPS subunit biosynthesis, steps related to EPS polymerization and export have been well defined in *R. leguminosarum* bv. *trifolii*, which allows proposing a robust model for exopolysaccharide secretion (see: [30] for review). In this Wzx/Wzy-dependent pathway, EPS subunits are transferred from the cytoplasm to the periplasm by Wzx-type flippase PssL and polymerized by Wzy-type polymerase PssT. The length of the growing EPS polymer is determined by polysaccharide co-polymerase PssP. PssN, i.e., an outer membrane homooligomeric lipoprotein, is a translocase allowing the export of the polysaccharide chain outside the cell [30]. Interestingly, other proteins encoded outside the Pss-I region also affect EPS production. A mutant in the *pssP2* gene localized within the Pss-II region in *R. leguminosarum* bv. *trifolii* [14] produced more EPS than the wild type strain, with predominating HMW fractions with molecular mass exceeding respective ones in the wild type strain [37]. PssP2 has been shown to interact with glycosyltransferase PssC and PssP/PssT proteins. Thus, it has been hypothesized that two similar co-polymerases, PssP and PssP2, may play complementary/opposite roles in determining the EPS polymerization degree, and PssP2 could be especially engaged in the polymerization of the LMW EPS fraction [37].

In search of an answer to the question of the ambiguous GTs involved in the biosynthesis of EPS, the function of the *pssJ* gene located in the Pss-I cluster of *R. leguminosarum* bv. *trifolii* TA1 (RtTA1) was investigated in the present study. A Δ*pssJ* deletion mutant was constructed and shown to produce EPS devoid of galactose. The *in trans* delivery of the *pssJ* gene restored galactosyltransferase activity. The impact of the *pssJ* mutation on the LPS production, symbiotic activity, motility, and stress sensitivity was shown. The postulated role of PssJ in the EPS “glycosyltransferase complex” was discussed in light of the results of the investigations of the subcellular localization and protein-protein interactions with other glycosyltransferases.

## 2. Results

### 2.1. PssJ Is Homologous to Galactosyltransferases

**Figure 1:**
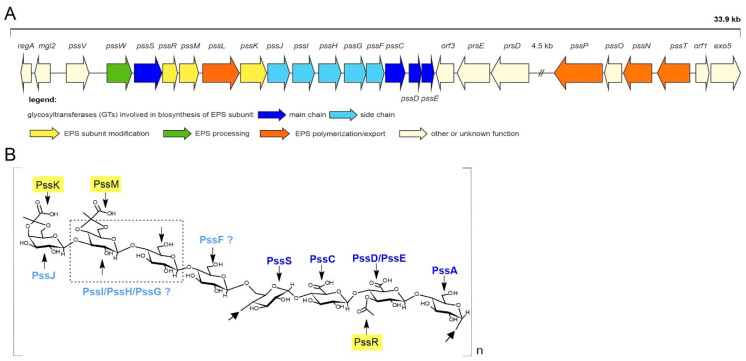
Genetic organization of the Pss-I gene cluster associated with biosynthesis of exopolysaccharide produced by *R. leguminosarum* bv. *trifolii* strain TA1 (**A**) and the biological structure of the exopolysaccharide (EPS) repeat subunit (**B**). Linkages, positions of acetyl and pyruvyl substituents, and gene products responsible for adding each sugar or non-carbon modification are shown. The sites of linkage of the backbone were marked with arrows. Colors for glycosyltransferases (GTs) in the map and the structure match. Genes that were previously missing in the RtTA1 Pss-I region are *pssH* and *pssG* being part of the cluster of genes encoding side-chain GTs.

Based on the previously available sequence of the Pss-I in the RtTA1 strain (accession no. DQ384110), the region comprised seven genes encoding hypothetical or experimentally verified glycosyltransferases engaged in the assembly of the EPS octasaccharide subunit. The region has recently been re-sequenced and annotated (accession no. MH595616), which revealed the presence of two previously unidentified genes: *pssG* and *pssH* (Figure 1). The gene named *pssH* in the previously submitted sequence was in fact *pssF*. Taking into account the newly established organization, the Pss-I region altogether comprises nine genes encoding glycosyltransferases (Figure 1). *pssA* gene encoding the so-called priming glycosyltransferase is located approx. 125 kb apart from the Pss-I cluster on the RtTA1 chromosome.

**Figure 2:**
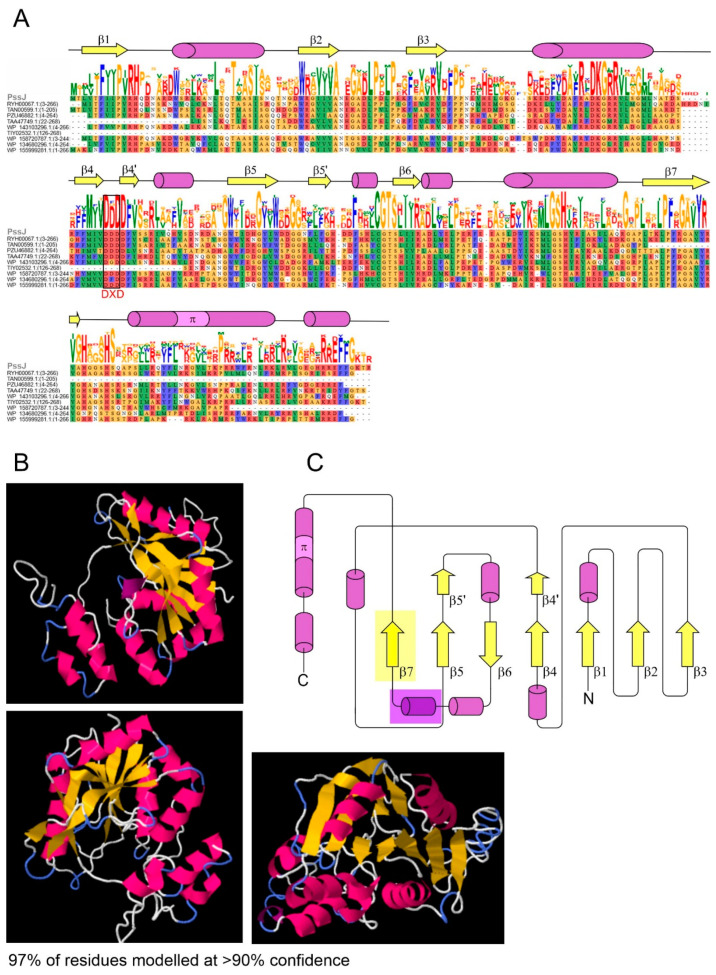
PssJ structure modeling. (**A**) Multiple alignment of amino acid sequences of PssJ homologs listed in Table S1 (the PssJ sequence covers amino acids 1–699). Predicted secondary structures are shown above the sequence logo. β-strands are shown in yellow and helices are marked in purple and pink. The frame indicates the position of the DXD motif found in GT-A glycosyltransferases. The ribbon diagram of the PssJ structure modeled with Phyre2 is shown in (**B**) and the secondary structure topology based on this and other secondary structure predictions is shown in (**C**). The H-DROP domain linker prediction indicated the presence of a linker between 178–197 aa in the PssJ sequence (purple shadow). No typical transmembrane segments were predicted, in contrast to the region of membrane interaction/association (yellow shadow).

One of the genes preceding the above-mentioned *pssI*-*pssH*-*pssG*-*pssF* cluster and together forming a block of genes with the hypothetical function of side-chain GTs is 822 bp *pssJ* (Figure 1) predicted to encode a galactosyltransferase (273 aa) responsible for the attachment of the last galactose residue to the octasaccharide subunit. ProtBLAST/PSI-BLAST similarity searches clearly indicated that the hypothetical PssJ could be the EPS terminal galactosyltransferase in RtTA1 (Supplementary Materials Table S1). Enzymatic activity responsible for this transfer was previously proposed based on the phenotype of the *exo344*::Tn5 mutant in *R. leguminosarum* bv. *trifolii* RBL5515 [9]. However, since then, the strain and the gene have not been further characterized. The PssJ homologues found were aligned, aiding in further prediction of the secondary structure and homology modeling of PssJ (Figure 2).

Based on the multiple sequence alignment and secondary structure predictions as well as homology modeling performed for the hypothetical PssJ galactosyltransferase with the use of Phyre2, a structure typical for GT-A glycosyltransferases was inferred for this protein, with a characteristic and highly conserved DXD motif, in which aspartate residues play a role in metal ion coordination. Its role is to stabilize charged phosphate groups of the nucleotide sugar donor substrate. The predicted secondary structures of PssJ shown in Figure 2 fold into an α/β/α sandwich with a seven-stranded β-sheet with 3214657 topology, in which strand 6 is antiparallel to the rest. Based on the template structures listed in Table S2, a PssJ model was predicted with a β-sheet forming a central cavity. The H-DROP domain linker prediction indicated the presence of a domain linker between 178–197 aa in the PssJ polypeptide, dividing the protein into two domains: an N-terminal Rossman-like domain with an active site and a C-terminal domain with a predicted β-strand and helices most probably involved in acceptor substrate binding and membrane interactions (Figure 2).

The predicted structure contains a typical β-sheet formed by six parallel and one anti-parallel β-strands, with helices surrounding this β-sheet core. Except for longer α-helices, one of the tools predicted the presence of a π-helix at the C-terminus of PssJ. Naturally occurring π-helices are typically short (7 to 10 residues) and are almost always flanked by α-helices on either end. Natural π-helices can easily be identified in a structure as a “bulge” within a longer α-helix, created by the insertion of a single additional amino acid into a pre-existing α-helix [38]. Neither signal sequences nor typical transmembrane helices were predicted for PssJ. However, a region of membrane association/interaction was predicted (Figure 2).

### 2.2. PssJ Is a Likely Biologically Active Galactosyltransferase Influencing EPS Biosynthesis

**Figure 3:**
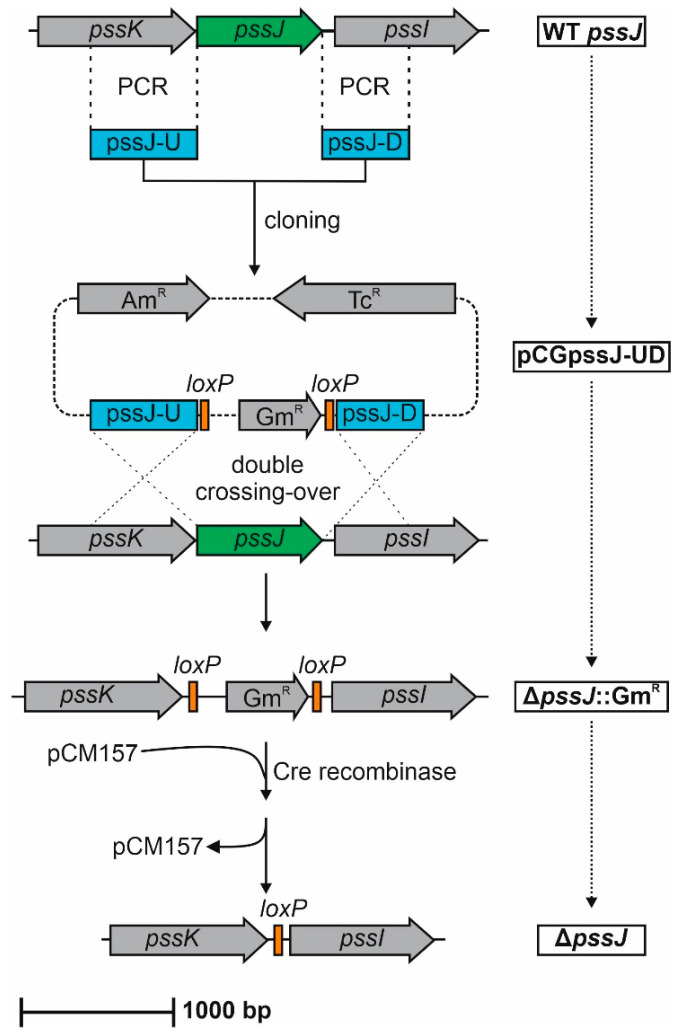
Schematic representation of the steps in the construction of the Δ*pssJ* mutant of the RtTA1 strain. Details of the construction as well as plasmids used are described in Section 4.

In order to delineate the function of the *pssJ* gene in RtTA1, a mutant with deletion of the gene was generated using the pCM351 allelic exchange vector [39] according to the previously implemented method [40] (Figure 3). The phenotype of the obtained Δ*pssJ* mutant was rescued by the transfer of plasmid pBKpssJ-C carrying the *pssJ* gene under P*lac* in the pBBR1-MCS2 plasmid (the complemented strain will be named Δ*pssJ*(*pssJ*) from now on). In the other plasmid used for complementation of Δ*pssJ*, the gene was fused with six codons for histidine (named Δ*pssJ*(*pssJ-*his6) thereafter).

**Figure 4:**
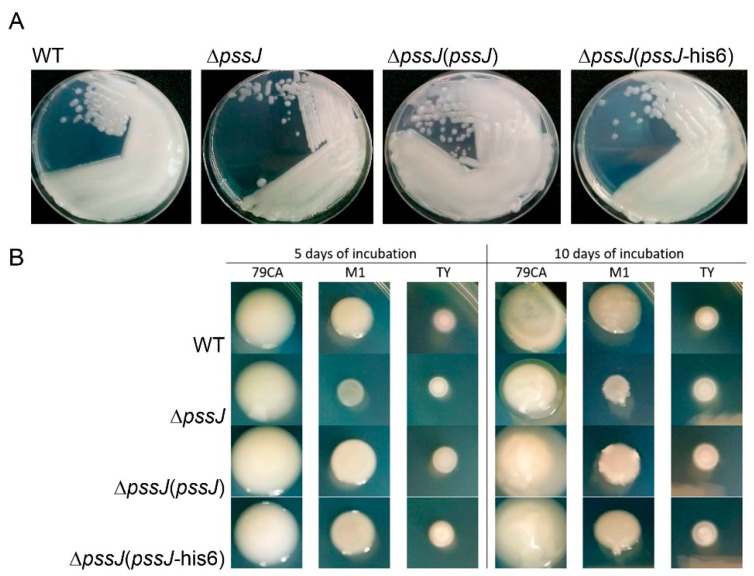
Single colony morphology (**A**) and growth/polysaccharide secretion (**B**) of the wild type strain and its derivatives: Δ*pssJ*, Δ*pssJ*(*pssJ*), and Δ*pssJ*(*pssJ*-his6) during cultivation in solid 79CA, M1, and TY media. Bacterial suspensions in the bottom panel were washed and standardized to the same starting optical density. Photographs were taken 5 and 10 days post-inoculation (left and right, respectively).

The macroscopic phenotype of the Δ*pssJ* mutant grown on the standard 79CA medium did not exhibit any significant differences from the wild type, except for the fact that the single colonies formed were smaller and less translucent (Figure 4). When analyzed in other routinely used media, Δ*pssJ* showed more differences in the growth kinetics and polysaccharide secretion. While in the 79CA complete medium, significant differences were visible after 10 days (e.g., a translucent halo in the case of Δ*pssJ*), a substantial defect in the growth/polysaccharide production by the Δ*pssJ* mutant was noticeable after 5 days in the minimal M1 medium, and the prolonged incubation (10 days) did not result in any compensation in polysaccharide secretion by the mutant. No differences were observed for the strains grown in the TY medium (Figure 4). These initial observations were further supported by the quantitative and qualitative analyses of EPS and LPS produced by the wild type strain RtTA1 and its derivatives, i.e., Δ*pssJ*, Δ*pssJ*(*pssJ*), and Δ*pssJ*(*pssJ*-his6).

**Figure 5:**
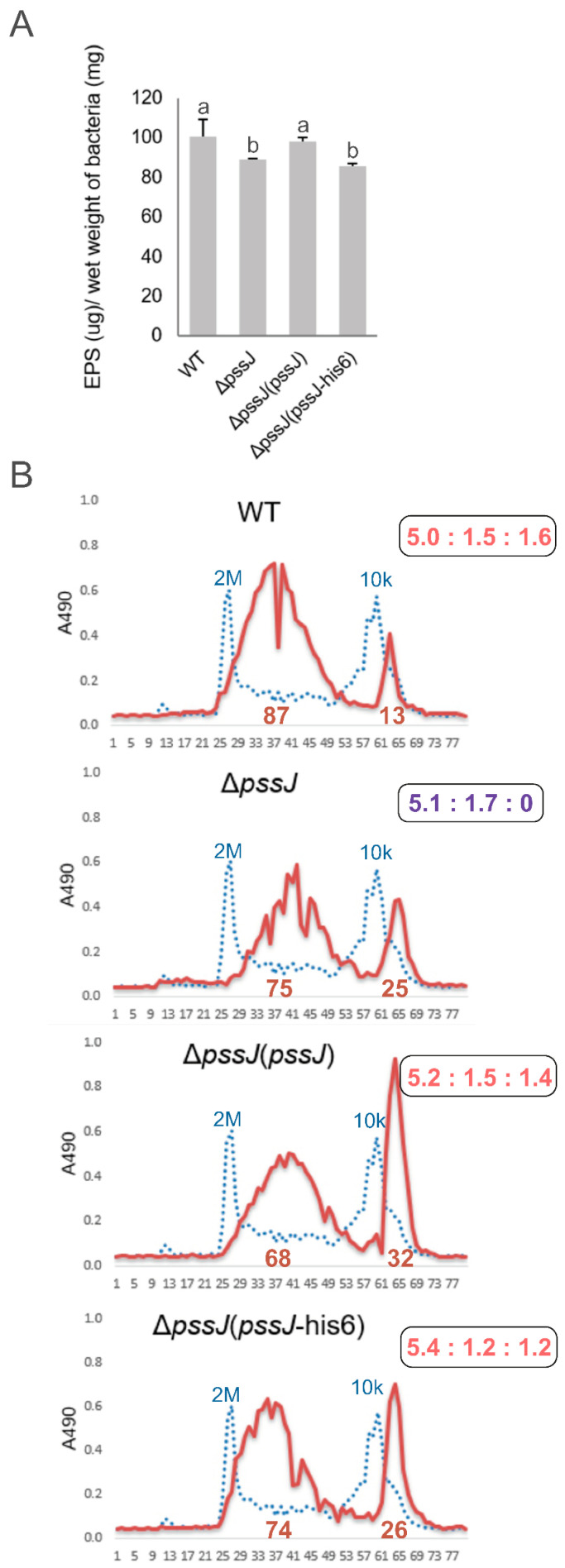
Exopolysaccharide production by the wild type RtTA1 and Δ*pssJ*, Δ*pssJ*(*pssJ*), and Δ*pssJ*(*pssJ*-his6) strains. (**A**) The total amount of EPS precipitated with 4 volumes of ethanol from the supernatants of cultures grown for 5 days in the 79CA medium containing 0.5% glycerol. Bars labeled with different letters represent values that are significantly different at *p* < 0.05. The amount of EPS is expressed as glucose equivalents. (**B**) Gel permeation chromatography of exopolysaccharides; the retention times of dextran blue (2 MDa) and dextran T10 (10 kDa) (molecular mass markers) are shown as blue dotted lines. Monosaccharide molar ratios in exopolysaccharides precipitated from supernatants are shown in the boxes on the right; the ratio specific for the mutant, indicating absence of galactose in EPS, is shown in dark blue.

The Δ*pssJ* mutant produced significantly less exopolysaccharide than the wild type (88.7 ± 0.6 vs. 100.6 ± 8.6 µg EPS/mg of bacterial pellet) (Figure 5A). The amount of total EPS produced was rescued in the complemented Δ*pssJ*(*pssJ*) strain (but not the one expressing His-tagged PssJ). The analysis of the hexose content revealed a lack of galactose in the acidic exopolysaccharide precipitated from the supernatant of the mutant culture, confirming the relation of PssJ with galactosyltransferase activity. The *in trans* delivery of the *pssJ* gene rescued the phenotype and the exopolysaccharide produced by the complemented strain contained galactose (Figure 5B). No concurrent changes in the LPS profiles in the RtTA1 derivatives were found (Figure S1). Size exclusion chromatography of the ethanol-precipitated material revealed no changes in the molecular weight of polymers in the HMW and LMW fractions. However, a pronounced change in the proportion between the fractions was observed in the case of the complemented strains. Both secreted more LMW material (Figure 5B).

### 2.3. The Lack of Terminal Galactose in EPS Affects the Symbiotic and Free-Living Traits of the Mutant

**Figure 6:**
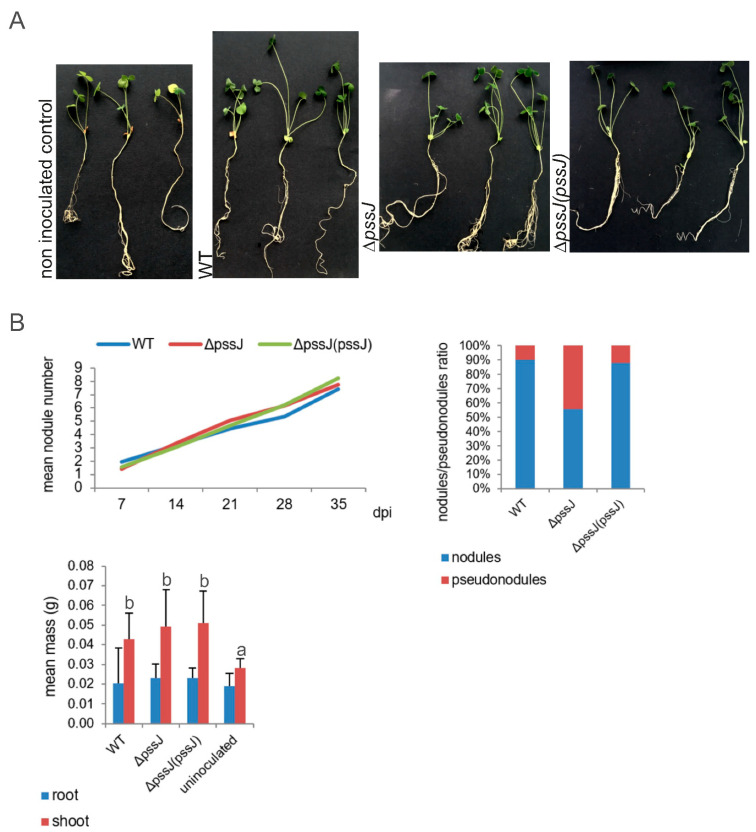
Symbiotic performance (**A**) and nodulation phenotypes (**B**) of wild type RtTA1 and its derivatives Δ*pssJ* and Δ*pssJ*(*pssJ*) on clover. Symbiotic performance was measured as nodulation kinetics throughout the experiment, number of nodules, and masses of shoots and roots. Significant differences in the shoot mass of clover infected with the wild type RtTA1 and its derivatives at *p* < 0.05 are marked with different letters above the standard deviation bars.

The symbiotic properties of *R. leguminosarum* bv. *trifolii* bacteria producing exopolysaccharide lacking terminal galactose were not substantially different from the wild type strain in terms of the kinetics of nodule formation, mean nodule number, and symbiotic nitrogen fixation measured as the green mass of clover shoots (Figure 6). Interestingly, the number of pseudonodules induced on clover roots by the Δ*pssJ* mutant was significantly higher in comparison to plants inoculated with the wild type or the complemented strain (Figure 6).

**Figure 7:**
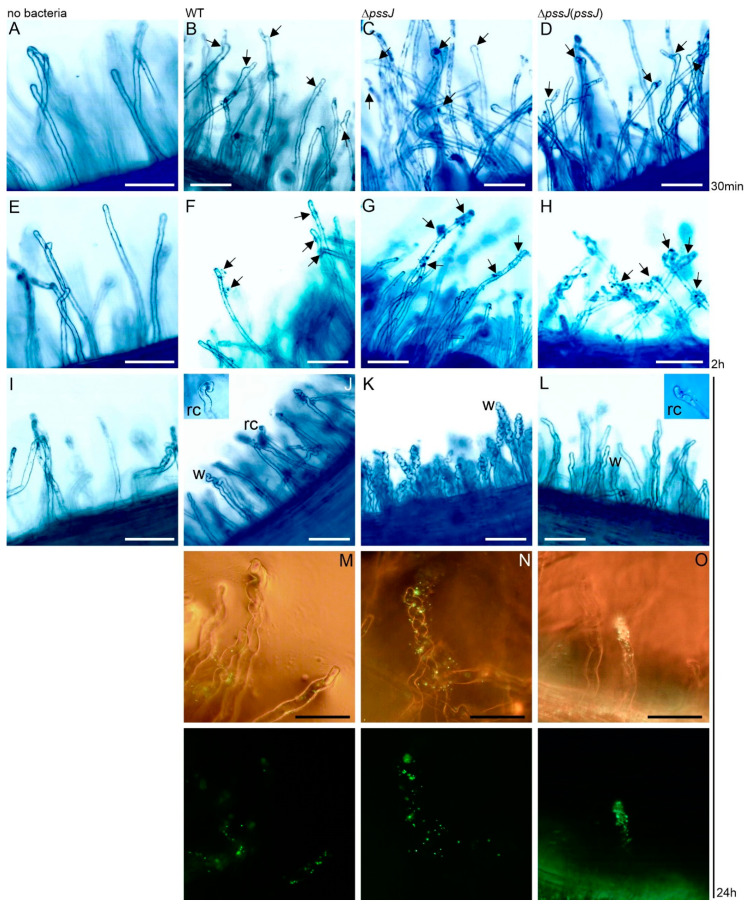
Light photomicrographs of root attachment and deformations induced on root hairs of *Trifolium pratense* L. cv. Nika after exposure to *R. leguminosarum* bv. *trifolii* TA1, the RtTA1Δ*pssJ* mutant, and its complemented derivative. Control seedlings were incubated in Fåhreus medium only (**A**,**E**,**I**). The arrows point towards sites of swelling and branching observed after 30-min incubation with the bacteria (**B**–**D**) and sites of bacterial attachment after 2 h of incubation (**F**–**H**). Panels (**J**–**O**) show root hairs after 24 h of incubation with the bacteria. Massive root hair wiggling (w) is clearly seen in the clover roots exposed to the mutant bacteria (**K**,**N**). In the case of the wild type (**J**,**M**) and complemented strain (**L**,**O**), wiggles (w) and typical root hair curls (rc) were observed. Fluorescence microscopy was used to visualize the GFP-expressing cells of the analyzed strains in the **last panel**. For each strain, six roots were examined. Bar = 50 µm.

To dissect the possible cause of such ineffective nodule infection by the mutant, we looked at the very first events leading to proper root hair deformation and entrapment of bacteria subsequently entering the plant cell trough infection threads. Clover seedlings were soaked in suspensions of the wild type, Δ*pssJ*, and Δ*pssJ*(*pssJ*) bacteria carrying the pMEG65 plasmid with the constitutively expressed *gfp* gene. Root hairs were inspected by light and fluorescence microscopy. After 30 min of incubation, root hairs exposed to all three strains showed signs of first deformations. However, in the case of Δ*pssJ*, most deformations involved only tip swelling, while the other two strains induced swelling and branching (Figure 7B–D). After two hours of incubation, all three strains adhered to the root hairs (Figure 7F–H) and, after 24 h, root hairs exposed to all the strains showed different types and degrees of deformation (Figure 7J–O). Only some hairs of the roots inoculated with the wild type strain were deformed and, besides tip wiggling, typical root hair curling was observed. Contrarily, roots inoculated with the mutant showed massive root hair wiggling and almost no typical curling. Root hairs exposed to the complemented strain were rarely wiggled but typical root hair curls were easily found (Figure 7J–O).

**Figure 8:**
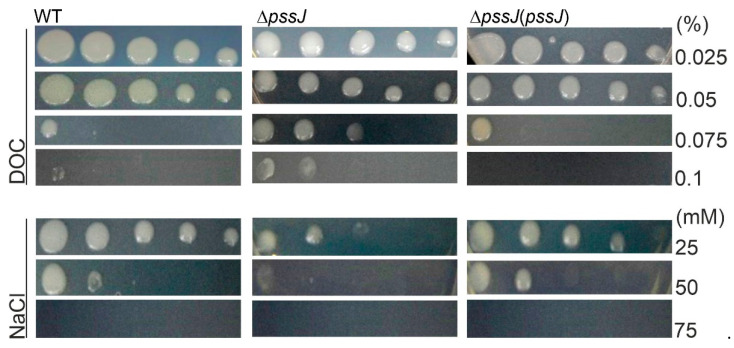
Influence of envelope stressors on the growth of the wild type RtTA1, Δ*pssJ*, and Δ*pssJ*(*pssJ*). Sensitivity to deoxycholate (DOC) and NaCl was determined by plating serial 10-fold dilutions of the bacteria on 79CA supplemented with the indicated concentrations of stressors.

Changes in the polysaccharide composition, molecular mass, or proportions between LMW and HMW EPS have been previously shown to play roles in bacterial sensitivity to stress conditions. From among the stressors tested in this work, NaCl affected the Δ*pssJ* growth, and this effect was clearly visible at 25 mM NaCl. Contrary to this result, the Δ*pssJ* mutant was more resistant to deoxycholate (DOC) and grew even in the presence of the highest DOC concentration tested (0.1%) (Figure 8). SDS, ethanol, and different pH values of the medium (Table S3) did not have any effect on the bacterial growth. Hydrogen peroxide had a clear effect on the mutant, and the diameters of the inhibition zones were 53.0 ± 1.0 mm, 58.0 ± 1.0 mm, and 51.0 ± 1.0 mm for the wild type, Δ*pssJ*, and Δ*pssJ*(*pssJ*), respectively.

**Figure 9:**
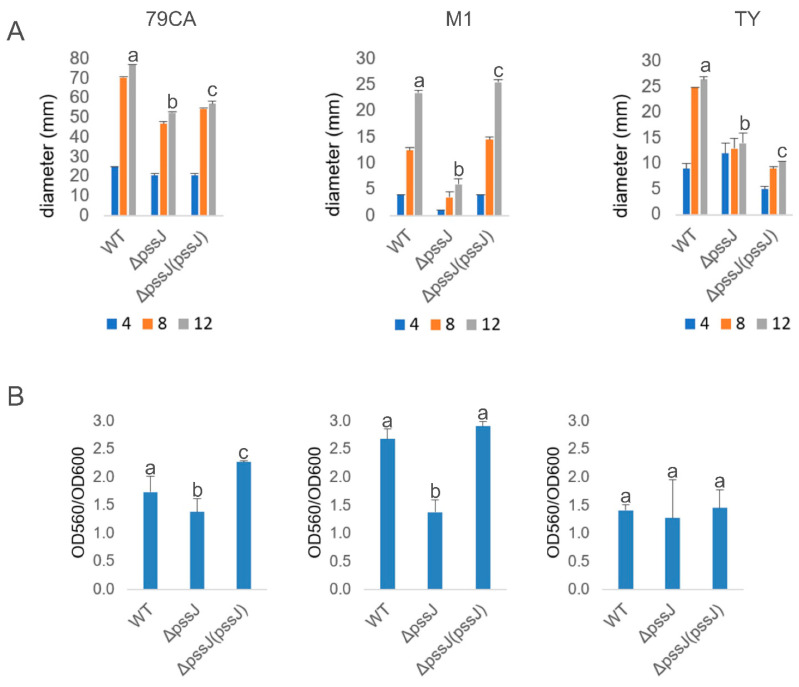
Swimming motility and biofilm formation of the wild type RtTA1 and its derivatives. Swimming motility was measured as the diameter of the zone around the puncture with bacterial suspension on days 4, 8, and 12 after inoculation into media solidified with 0.3% agar (**A**). Biofilm formation was examined by staining with crystal violet and expressed as the ratio of the amount of crystal violet solubilized by ethanol to the bacterial growth (OD_560_/OD_600_) (**B**). Bars of standard deviation labeled with different letters represent values significantly different at *p* < 0.05.

EPS biosynthesis is crucial for the biofilm-forming properties of bacteria and negatively correlated with swimming motility. The Δ*pssJ* mutant was significantly affected in swimming motility in all the tested media, but this defect was fully complemented by the *pssJ* gene delivery only in the case of the M1 minimal medium (Figure 9A). The amount of biofilm formed by the tested strains generally correlated with the amount of EPS produced. Both in 79CA and M1, the amount of biofilm measured with the crystal violet staining/destaining method was significantly lower and this defect was rescued in the complemented strain (Figure 9B).

### 2.4. PssJ May Play Its Role at the Membrane Interface by Interactions with Other Membrane GTs

PssJ was predicted to have neither signal peptides nor membrane-spanning regions. However, the predictions were not fully consistent, as several tools indicated inner membrane or periplasmic localization of PssJ, without predicting internal transmembrane (TM) helices. PSIPRED identified a membrane-interaction region (not the transmembrane segment) between 205–220 aa of the PssJ sequence (Figure 2).

The subcellular localization of the PssJ protein was analyzed in two ways. Firstly, anti-PssJ specific antibodies were obtained in chicken as an immunization host and used in the localization study. Secondly, a Δ*pssJ* mutant was complemented with a plasmid construct encoding a protein tagged with 6× His at the C-terminus. The functionality of the PssJ-His_6_ protein in RtTA1 cells was confirmed by the polysaccharide analyses described above (Figure 4 and Figure 5).

**Figure 10:**
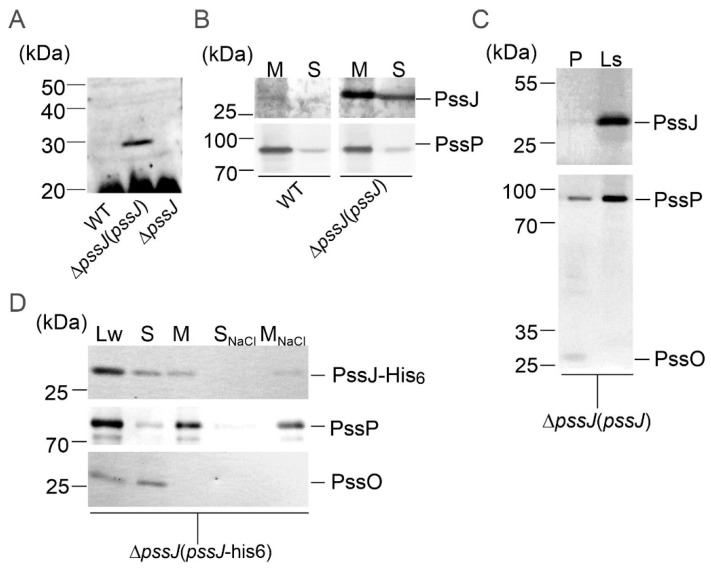
Localization of PssJ and PssJ-His_6_ proteins. (**A**–**C**) Western blotting with chicken anti-PssJ IgY affinity-purified antibodies to whole-cell lysates of the wild type strain, Δ*pssJ*(*pssJ*), and Δ*pssJ* (**A**); soluble (S) and membrane fractions (M) of the wild type strain and Δ*pssJ*(*pssJ*) (**B**); periplasmic proteins and lysate from the spheroplasts of Δ*pssJ*(*pssJ*) (**C**), Western blotting with mouse anti-His6 performed to whole cell lysate (Lw), soluble (S), membrane-containing (M), membrane-associated (S_NaCl_), and integral membrane (M_NaCl_) protein fractions of the Δ*pssJ*(*pssJ-*his6) strain (**D**). Secondary antibodies conjugated with horseradish peroxidase were used, which was followed by chemiluminescent detection (**A**). PssO is a soluble periplasmic protein and PssP is a typical transmembrane protein with two TMS. Both served as fraction purity markers. Loading was standardized to the volume of lysate, allowing visual assessment of the cellular distribution of PssJ.

Proteins from different cell types, i.e., RtTA1, Δ*pssJ*, Δ*pssJ*(*pssJ*), and Δ*pssJ*(*pssJ*-his6), were fractionated and probed for the presence of PssJ or PssJ-His_6_. PssJ was repeatedly hardly detectable with chicken anti-PssJ antibodies in the wild type strain, while it was clearly visible in the case of the Δ*pssJ*(*pssJ*) bacteria. The intensity of PssJ bands in both strains were dramatically different in samples containing the same concentration of total proteins, showing that the level of native PssJ in the WT cells is very low in comparison to the complemented strain, in which untagged PssJ was easily detected (Figure 10A,B). In Δ*pssJ*(*pssJ*), PssJ was present both in the soluble and total membrane protein fractions (Figure 10B). However, the presence of PssJ in the periplasm was ruled out by probing periplasmic proteins and total spheroplast lysate obtained from the Δ*pssJ*(*pssJ*) strain with the anti-PssJ antibodies (Figure 10C). PssJ-His_6_ was present in both the soluble and total membrane protein fractions and washing the membrane samples with 1 M NaCl did not solubilize PssJ-His_6_ (Figure 10D). The results did not give unambiguous evidence for its presence either in the cytoplasm or in the inner membrane. Rather, the data indicate that PssJ plays its role at the membrane interface.

Since most of the GTs involved in EPS subunit synthesis in *R. leguminosarum* bv. *trifolii* are predicted to be inner membrane proteins, we intended to check whether the PssJ “membrane interaction” might result from its interactions with other GTs. To this end, a bacterial two-hybrid system (BTH) was employed. All ten genes encoding glycosyltransferases were cloned into four vectors of the bacterial two-hybrid system and used in systematic co-transformations and interaction screening in the reporter strain *Escherichia coli* DHM1. Here, we also used previously constructed plasmids carrying genes encoding the PssT, PssP, PssL, and PssP2 proteins, i.e., the components of the Wzx/Wzy-dependent system.

**Figure 11:**
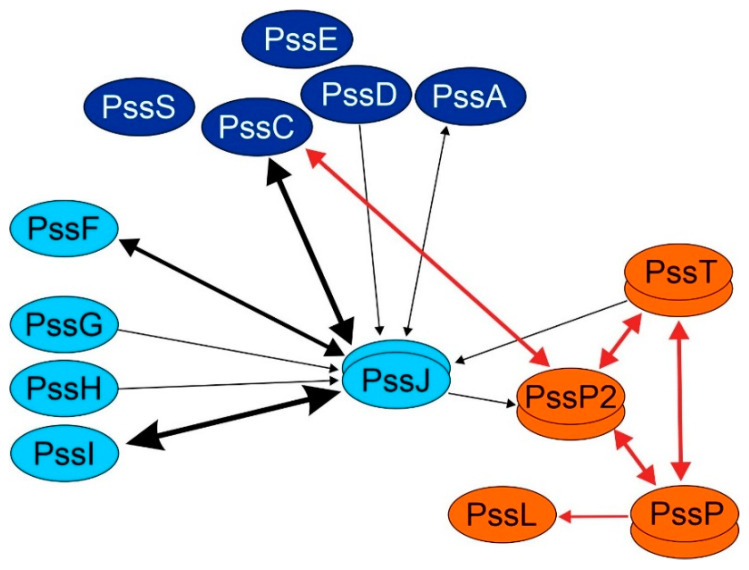
Map of interactions between PssJ, other GTs, and Wzx/Wzy-system proteins based on the bacterial two-hybrid results. Proteins in dark blue—GTs involved in the transfer of sugars in the main chain of the subunit; proteins in pale blue—involved in the synthesis of the side chain, and orange—flippase PssL, co-polymerases PssP/PssP2, and polysaccharide polymerase PssT (colors match those in Figure 1). The thickness of the line linking the two proteins represents the number of “positives” identified in 4–8 combinations of co-transformed plasmids encoding GTs and other Pss proteins. The arrows point towards bait proteins expressed from the pUT18/pUT18C plasmids. Black lines—interacting pairs identified in this work; red lines—interacting pairs identified in previous works [37,41].

Based on the dense map of interactions obtained, we concluded that PssJ is engaged in contacts with both GTs involved in main-chain and side-chain synthesis. PssC glucuronosyltransferase (main chain) as well as PssF and PssI glucosyltransferases (side chain) seem to be the two most important partners (Figure 11). The other Pss proteins tested gave no or single positive combinations. In the case of the PssT and PssP2 proteins, it was previously shown that not all combinations were fully functional, which in combination with the high hydrophobicity and molecular mass of these integral membrane proteins may have contributed to only single positive pairs PssJ-PssT and PssJ-PssP2 (Figure 11). Two out of four combinations for homotypic PssJ-PssJ interactions were positive, thus the protein, like other GTs, may form at least dimeric forms.

## 3. Discussion

Exopolysaccharide synthesis in rhizobia is governed by proteins classified as components of the Wzx/Wzy-dependent system. In the case of such heteropolysaccharides, an oligosaccharide lipid-linked subunit is synthesized first and then translocated to the periplasmic side of the inner membrane, where polymerization occurs. Glycosyltransferases responsible for EPS subunit assembly and enzymes decorating EPS with non-carbohydrate substituents have been partially characterized in *R. leguminosarum* bv. *trifolii* (for review see: [30]). In the case of glycosyltransferases, most information concerns GTs engaged in the assembly of the main chain of the EPS subunit [24,27,28,29]. As to the side chain glycosyltransferases, the only data refer to analyses of a putative terminal galactosyltransferase. A *R. leguminosarum* bv. *trifolii* RBL5515 strain carrying the mutation known as *exo344*::Tn5 was described previously [9]. The mutant produced only residual amounts of EPS, whose repeating units lacked terminal galactose in the side chain. The results of the enzyme activity and structural analyses of the polysaccharides synthesized by the mutant led to the conclusion that the mutation disrupted a hypothetical gene coding for galactosyltransferase responsible for the addition of the terminal galactose residue in the octasaccharide unit. However, no DNA sequencing data confirming the location of the *exo344*::Tn5 insertion were provided and the gene was not characterized.

The data provided in this work support the conclusion that *pssJ* located in the Pss-I gene cluster in fact encodes a galactosyltransferase responsible for the attachment of terminal galactose to the EPS repeat subunit in the *R. leguminosarum* bv. *trifolii* TA1 strain.

Difficulties with high-level expression, purification, and crystallization hampered determinations of the crystal structure for GT enzymes for a long time. However, the number of available structures of GTs available in the RCSB Protein Data Bank exceeded 2500; therefore, much more reliable homology modeling for putative GTs is possible now. The predicted secondary and tertiary structures of the PssJ protein resemble the GT-A fold found in many glycosyltransferases. The GT-A fold consists of an α/β/α sandwich with a seven-stranded β-sheet resembling a Rossmann fold [19]. Depending on GT, the two aspartate amino acids are not always conserved, but this particular motif, or its variants, can always be identified at the same location, i.e., in a short loop connecting one β-strand of the main α/β/α sandwich to a smaller one [42]. In the modeled PssJ structure, the central β-sheet is indeed flanked by a smaller one, and a common DxD motif is present inside this cavity, which supports the PssJ requirement for a divalent cation for activity, as in the case of other GT-A enzymes [43,44].

The subcellular localization of the PssJ protein is not unequivocal. Online tools indicated three of the four compartments possible in the Gram-negative bacterial cell (except the outer membrane) as a probable destination for PssJ, and no typical transmembrane helices were detected in the amino acid sequence. PssJ, either native or His_6_-tag-equipped, was found in soluble and membrane protein fractions. GTs are membrane-associated or hydrophobic integral membrane proteins [19]. The localization and interaction study conducted in this work indicates that PssJ may be a dimeric membrane-associated protein, which results from direct interactions of certain structures with the inner membrane or interactions with typical integral membrane proteins, i.e., other glycosyltransferases and proteins of the Wzx/Wzy-dependent system. Given the detected heterotypic interactions with PssF and PssI GTs, probably involved in side-chain synthesis, and PssC GT involved in the main-chain synthesis, all of which are predicted to possess transmembrane segments, it becomes obvious that PssJ may be present in the membrane fraction as part of putative GTs complex/subcomplexes, although it is not an integral membrane protein itself. Previously, interactions between the polymerization/translocation machinery and glycosyltransferases were detected [41]. The single positive pairs PssJ-PssT and PssJ-PssP2 detected in the BTH screening in this work do not undoubtedly support they are true positives. However, taking into account the role of PssJ as a terminal galactosyltransferase, contacts between GT adding the last sugar residue to the EPS subunit and proteins/enzymes acting afterwards, i.e., PssL, PssT, or PssP2, cannot be excluded. The ambiguous results obtained in the BTH assay related to the interaction of PssJ and the components of the Wzx/Wzy system may also indicate the temporary character of such interplay.

An irrefutable proof for the role of PssJ in galactose transfer in EPS subunit synthesis is the sugar content of the exopolysaccharide secreted by the Δ*pssJ* mutant. No galactose was detected in the material precipitated from the mutant culture supernatant, while it was present in case of the wild-type strain and both complemented derivatives. *pssJ* deletion results in a significant decrease in the amount of secreted EPS. However, the present results have clearly shown that the lack of the terminal galactose (and probably the non-sugar substituent normally attached to it) in the mutant does not block further stages of EPS subunit processing. It may still be recognized by PssL and flipped into the periplasm, polymerized by PssT/PssP/PssP2 proteins, and translocated outside the cell via a PssN translocon. The molecular mass of polymers was not changed in the mutant either. However, the increase in the amount of the LMW fraction in the mutant and both complemented strains suggests that the efficiency of polymerization of EPS is probably affected by the loss-of-function of PssJ in the mutant and changes in the *pssJ* gene dosage in the complemented bacteria. Overexpression of *pssJ* directed by P*lac* may affect the effective incorporation of ready subunits into the growing chain of polysaccharide, even though they are flipped on the periplasmic side of the inner membrane (or there may be a bottle-neck at the PssL translocation step; when flipping is less efficient than normal, inefficient HMW polymerization and release of short oligosaccharides may consequently occur [34]). The latter may also result from disturbances in the stoichiometry of protein subcomplexes.

A higher level of the LMW fraction (seen also as a translucent halo after prolonged culturing in the 79CA medium) may also represent cyclic β-glucans, an increased amount of which in culture supernatants may result from enhanced leakage from the periplasmic space [45]. The *exo344*::Tn5 mutant, besides the lack of galactose, was characterized by a 7-fold decrease in EPS production and a 7-fold increase in the concentration of cyclic glucans in the supernatant. In the case of RtTA1 Δ*pssJ*, these changes were less pronounced. It was previously shown that LMW fraction contains monomers of EPS subunits [9]. In the case of the Δ*pssJ* and complemented strains, this would indicate the above-mentioned influence on the performance of the polymerization system.

Interestingly, the consequences of the lack of galactose and its substituents in EPS on the symbiotic performance of the Δ*pssJ* mutant were reflected in the increased number of round, white, ineffective nodules on clover roots. Neither the nodulation kinetics nor the mass of shoots differed from the wild type, indicating that nitrogen fixation was not disturbed. Surprisingly, no nodulation was observed in the case of the *exo344*::Tn5 mutant [9]. The increase in the number of pseudonodules, without an effect on the overall symbiotic interaction, indicated that some interference at the level of clover EPS receptor–modified EPS of Δ*pssJ* or the nodule infection stage may have occurred. Nod factor (NF) perception is indispensable for infection and nodule organogenesis, but exopolysaccharides and cyclic glucans have been demonstrated to modulate the infection process. Kawaharada et al. [6] have shown that the expression of the EPS receptor is inducible by NF and sufficient to select compatible bacteria through the recognition of their exopolysaccharide. Thus, a two-stage mechanism involving recognition of both NF and EPS has been proposed [46]. EPSs have been postulated to participate in the suppression of defense responses, allowing rhizobia to progress toward cortical cells without triggering hypersensitive response [47]. The Δ*pssJ* mutant is less motile and exhibits disturbed biofilm formation, especially in a growth-limiting medium. It was also slightly more sensitive to increased salt and ROS levels, but slightly more resistant to DOC. This may result from the changes in the physicochemical features of EPS devoid of galactose and non-sugar decorations normally attached to it and the elevated content of LMW oligosaccharides. Specific binding of EPS to the receptor is important for proper recognition of compatible bacteria and their entrapment [30]. Thus, if Nod factor signaling important for the formation of nodule primordia in the first stage is not affected, disturbed recognition of bacteria producing EPS with a changed structure in the second stage, followed by a decrease in nodule infection, may be the cause of the formation of a large number of empty nodules. The possible causes of the disturbed nodule infection include the slightly increased susceptibility of the Δ*pssJ* mutant to higher salt and reactive oxygen species levels, which may be encountered after release inside plant cells, and/or increased plant defense response as a result of the recognition of the changed EPS structure.

## 4. Materials and Methods

### 4.1. Bacterial Strains and Standard Culture Conditions

Bacterial strains and plasmids used in this work are listed in Table 1. *E. coli* strains were grown in lysogeny broth (LB) medium at 37 °C [48] and *R. leguminosarum* bv. *trifolii* strains were grown in 79CA with 1% mannitol or 0.5% glycerol at 28 °C [49], tryptone–yeast (TY) [50], or M1 [51] media. *E. coli* DHM1 reporter strain was grown at 30 °C. Antibiotics were used at following final concentrations: 100 μg/mL ampicillin, 40 μg/mL kanamycin, 5 (*E. coli*) or 10 μg/mL (*Rhizobium*) gentamicin, 10 μg/mL tetracycline, and 40 μg/mL rifampin.

### 4.2. Bioinformatic Analyses

Sequence data were analyzed with Lasergene analysis software (DNASTAR, Inc., Madison, WI, USA). Putative homologues of PssJ were identified with ProtBLAST/PSI-BLAST [58]. Top results not described as “hypothetical” were aligned using ClustalΩ [59] and forwarded to Alignment Viewer. All these are available at https://toolkit.tuebingen.mpg.de/. Protein homology modelling was performed with Phyre2 [60]. Secondary structure predictions were performed with Predict Protein [61], Jpred4 [62], RaptorX [63], and PSIPRED [64]. H-DROP [65] was used to predict domain linkers. Prediction of signal sequences, transmembrane segments, and subcellular localization was done with: CCTOP [66], PRED-TAT [67], PSORTb [68], Phobius [69], TMHMM [70], and Gneg-mPloc [71].

### 4.3. DNA Techniques

DNA isolation was performed with Plasmid Mini and Genomic Mini kits according to manufacturer’s protocols (A&A Biotechnology, Gdynia, Poland). Molecular cloning and transformation were performed according to standard protocols [53] Volume 1, Chapter 1, 1.84–1.87 and 1.116–1.118. Fast Digest restriction endonucleases were purchased in Thermo Fisher Scientific (Waltham, MA, USA). Plasmids and primers used in this work are listed in Table 1 and Table S5, respectively. PCR was performed with high-fidelity Platinum SuperFi DNA polymerase (Thermo Fisher Scientific, Waltham, MA, USA). Sanger DNA sequencing of plasmid constructs prepared in this work was performed in Genomed (Warsaw, Poland).

### 4.4. Construction of the ΔpssJ Mutant and PssJ Complemented Strains

The regions flanking *pssJ* were amplified by PCR using RtTA1 genomic DNA as a template. The purified 700-bp PCR product for *pssJ* upstream region was cloned into NdeI site of pCM351 to produce pCGpssJ-U. Subsequently, the purified 570-bp PCR product for *pssJ* downstream region was introduced between ApaI–SacI sites of pCGpssJ-U, resulting in pCGpssJ-UD (Figure 3). pCGpssJ-UD was transferred to RtTA1 by biparental conjugation from *E. coli* S17-1 [52]. Bacterial mating experiments were performed as described by Reeve et al. [72]. Gentamicin-resistant transconjugants obtained on TY medium containing rifampicin and gentamicin were subsequently screened for tetracycline sensitivity in order to identify potential null mutants. The frequency of double-crossover events was 4.5%. One such mutant was chosen for further study (TA1Δ*pssJ*(Gm^R^)). Analytical PCRs confirmed the successful allelic exchange. To remove the gentamicin resistance cassette, the plasmid pCM157 was introduced into TA1Δ*pssJ*(Gm^R^) by electrotransformation, as described by Garg et al. [73]. Tetracycline-resistant transformants were streaked for purity by two passages to obtain strain called TA1Δ*pssJ*[pCM157], which produced only gentamicin-sensitive colonies. pCM157 was cured from TA1Δ*pssJ*[pCM157] by five consecutive transfers on medium lacking tetracycline to obtain the Δ*pssJ* mutant strain. Analytical PCRs were performed in order to confirm successful deletion of the gentamicin resistance cassette. The sequencing of the PCR-amplified product indicated expected recombination between *loxP* sites. For the construction of pBK*pssJ*-C plasmid used in complementation analyses, a 990-bp PCR fragment comprising *pssJ* was cloned between ApaI–XbaI sites of pBBR1-MCS2 under P*lac* of the vector. In order to construct C-terminally His6-tagged version of PssJ, a 940-bp fragment comprising *pssJ* gene equipped with in-frame 3′-terminal His6-tag coding sequence was cloned into ApaI–XbaI sites of pBBR1-MCS2, resulting in pBK*pssJ*-C-His6. Obtained plasmids were transferred into Δ*pssJ* mutant cells through electrotransformation.

### 4.5. Exopolysaccharide and Lipopolysaccharide Analyses

Extracellular polysaccharides were precipitated with 4 volumes of ethanol from the supernatants of bacterial cultures grown with shaking for 5 days in 79CA medium with 0.5% glycerol. Exopolysaccharides were subjected to gel permeation chromatography on a Sepharose CL-6B column (1.0 cm × 90 cm) (Sigma-Aldrich, Saint Louis, MO, USA) using 1M NaOH as eluent and a gravity flow at 0.2 mL/min. Blue Dextran, 2 MDa, and Dextran T10, 10 kDa were used as molecular mass standards. Total sugar content was determined according to Dubois et al. [74] and calculated in glucose equivalents. The glycosyl composition of EPSs was analyzed as described previously [37] by preparation of the alditol acetate derivatives, identified and quantified by gas liquid chromatography mass spectrometry (GLC-MS). In brief, EPS samples were hydrolyzed with 2 M CF_3_CO_2_H at 100 °C for 4 h, reduced with NaBD_4_, and acetylated with a 1:1 (*v*/*v*) mixture of acetic anhydride and pyridine (85 °C, 0.5 h). To confirm the presence of uronic acids, methanolysis, and carboxyl reduction with NaBD_4_ prior to hydrolysis was performed. Monosaccharides converted into alditol acetates were analyzed with an Agilent Technologies 7890A gas chromatograph (USA) connected to a 5975C MSD detector (inert XL EI/CI, Agilent Technologies, Santa Clara, CA, USA). The chromatograph was equipped with an HP-5MS capillary column (Agilent Technologies, 30 m × 0.25 mm, flow rate of 1 mL min^−1^, He as the carrier gas). The temperature program for the derivatives was as follows: 150 °C for 5 min, then 150 to 310 °C at 5 °C min^−1^ and the final temperature was maintained for 10 min. Lipopolysaccharides were isolated by whole-cell microextraction as described by Apicella [75], analyzed in Tricine SDS-PAGE, and visualized by silver staining according to Tsai and Frasch [76].

### 4.6. Plant Tests

Seeds of red clover (*T. pratense* L. cv. Nike) were surface sterilized with sublimate (0.1% mercury chloride solution) and germinated in Petri dishes with a nitrogen-free plant medium [77]. Two-day-old sprouts were transferred to the slants and left for 4–5 days to grow the first cotyledons. After this time, the plants were inoculated with 0.1 mL bacterial suspension at OD_600_ of 0.1. Plants were grown in a greenhouse (14/10 h, day/night). After 6 weeks, the plants were harvested and the masses of fresh shoots and roots were measured. The number of root nodules formed were checked at weekly intervals for 6 weeks after plant inoculation. The experiment was carried out in 30 replicates for each strain.

### 4.7. Root Attachment Assay

Assays for root attachment of RtTA1, Δ*pssJ*, and Δ*pssJ*(*pssJ)* carrying pMEG65 with a constitutively expressed *gfp* [57] were done with the strains grown in 79CA solid medium. Bacteria were washed twice with sterile water and suspensions in Fåhraeus nitrogen-free medium [77] were prepared (OD_600_ of 0.1). Sterile germinated clover seedlings with ≈2 cm long root and visible root hair zones were soaked in respective suspensions. After 0.5, 2, and 24 h of incubation, seedlings were transferred to a tube containing sterile water and vortexed 3 times for 5 sec with each wash, and finally soaked in sterile water before microscopic observations. For light and fluorescence microscopy Olympus BX41 microscope equipped with OlympusUSH-103OL mercury lamp was used.

### 4.8. Examination of Sensitivity to Stress Factors

To determine the sensitivity to envelope stressors, bacteria were cultured for 48 h in liquid 79CA medium with 1% mannitol as a carbon source. The cultures were then diluted to OD_600_ of 0.2, 1 mL of suspension was centrifuged at 6000× *g* with cooling for 10 min, and washed once with sterile water. A series of 5 subsequent 10-fold dilutions was prepared, of which 2.5 μL was spotted onto 79CA medium supplemented with the appropriate concentration of stress factor: SDS (0.01–0.05% *w/v*), DOC (0.025–0.1% *w/v*), ethanol (1–5% *v/v*), and NaCl (25–100 mM). After the 4-day incubation at 28 °C, the growth was checked and photographed. Influence of medium pH was examined with identical bacterial suspensions spotted onto 79CA plates with pH adjusted to 7.2 (normal), 6.7, 6.2, 5.7, and 5.5.

### 4.9. Tolerance Against 30% Hydrogen Peroxide

For this experiment, a double-layer method was used. The 48-h-cultures in 79CA with 1% mannitol were diluted to OD_600_ of 0.2, 100 µL of suspension was added to 3 mL 79CA medium containing 0.35% agar, and poured onto solidified 1.5% 79CA agar plates. Paper disks (5 mm diameter) were applied to the center of the plate and 2.5 μL-drops of 30% H_2_O_2_ were poured onto. After 4 days of incubation at 28 °C, the growth inhibition zone was measured.

### 4.10. Motility Tests

To test the swimming motility, bacteria were cultured in liquid 79CA medium with shaking for 24 h. The cultures were diluted to OD_600_ of 0.4, 1 mL was centrifuged at 6000× *g* for 5 min, washed once with sterile water, and the obtained pellet was suspended in 1 mL of sterile water. The sterile tip/needle was immersed in bacterial suspension and punctured into 79CA, TY, and M1 solidified with 0.3% agar. Plates were incubated at 28 °C and growth/spread zone was measured after 4, 8, and 12 days of incubation.

### 4.11. Biofilm Formation Assays

Tested strains were cultured in liquid 79CA, TY, and M1 for 48 h with shaking. The cultures were then diluted with a corresponding medium to obtain OD_600_ of 0.1, 200 μL of each suspension was transferred to wells of 96-well microplate and incubated with shaking at 100 rpm for 72 h. Bacterial growth was determined by measuring OD_600_. Planktonic cells were gently removed and 150 μL 0.1% crystal violet was added to the wells and left for 1 h. Then, the wells were washed three times with water and 150 μL of 95% ethanol was added to each well and incubated with shaking (100 rpm) for 15 min at 28 °C. The optical density of dissolved crystal violet solution was measured at 560 nm using a Benchmark Plus™ plate reader (Bio-Rad Laboratories, Portland, ME, USA). The experiment was carried out in six replicates for each strain in parallel.

### 4.12. Preparation of His_6_-PssJ Inclusion Bodies

Several attempts to purify PssJ antigen from non-inclusion fraction of *E. coli* cells carrying pET30c-pssJ were unsuccessful (data not shown). Different culture conditions were tested for different expression hosts carrying pET30c-pssJ as well as other constructs made in pT7-7 (N- and C-terminal fusions) and pGEX4T-1 (data not shown). These obstacles were the main causes to use purified His_6_-PssJ inclusion bodies for immunization of hens instead of soluble protein (Figure S2). His_6_-PssJ protein was purified from *E. coli* BL21(DE3) cells carrying the expressing vector pET30c-pssJ (see: bacterial strains and plasmids). Cells were grown at 37 °C till OD_600_ of 0.8 and induced with IPTG at a final concentration of 0.5 mM. After 5 h of post-induction growth at 37 °C, cells were harvested and frozen at −20 °C. After thawing, cells were disrupted by sonication in PBS with 5 mM 2-mercaptoethanol and proteases inhibitors. Unbroken cells were pelleted by centrifugation (5 min, 4000× *g*, 4 °C). Supernatant was further centrifuged for 15 min, 10,000× *g*, 4 °C in order to separate soluble protein fraction from insoluble proteins. Inclusion bodies were washed twice with the same buffer by resuspending in a glass homogenizer and centrifuged as described above. Inclusion bodies were then suspended in 2 M NaCl, 6% Triton X-100, and left for 30 min on ice. After centrifugation as above, the pellet was solubilized in 100 mM NaH_2_PO_4_, 10 mM Tris-HCl, 6 M GuHCl (pH 8.0) at room temperature for 60 min. Aggregates were pelleted and the supernatant was subjected to purification on NiNTA resin according to the manufacturer’s protocol (QIAGEN, Hilden, Germany). Protein eluted from the resin was subjected to refolding through overnight dialysis against PBS with 5 mM 2-mercaptoethanol and proteases inhibitors or 25 mM Tris-HCl (pH 7.4), 150 mM NaCl, 10 mM MgCl_2_, 1 mM DTT, and protease inhibitors. No refolding was observed and 100% of protein precipitated from the solution. Precipitated protein was resuspended in PBS and used for immunization of hens.

### 4.13. Preparation of Anti-PssJ Specific Chicken Antibodies

Immunization of hens and isolation of IgY antibodies were performed as follows. Four 22-week-old White Leghorn egg-laying hens were purchased from a commercial source (Woźniak Poultry Farm, Żylice, Poland). Antigen in a form of His_6_-PssJ inclusion bodies was suspended in 0.9% saline (2 mg/mL) and sonicated for 20 min. Antigen was emulsified with Freund’s complete adjuvant (1:1, *v/v*). Animals were immunized intramuscularly at two different sites with 200 μL per site (150 μg/animal). The booster injections were administered after 5, 8, and 13 weeks of primary immunization (75 μg/animal) in Freund’s incomplete adjuvant. The isolation of IgY antibodies from eggs collected daily was conducted separately for each egg according to the procedure described previously [78] with minor modifications [79]. Western blot and ELISA were used to examine the development of immune response over the course of hens immunization (Figure S3). Anti-PssJ antibodies were purified from crude isolates by affinity chromatography (Figure S4) according to the protocol described previously [80]. Affinity-purified antibodies were used in Western blotting at a final concentration of 1 µg/mL.

### 4.14. Rhizobium Cells Fractionation and Analyses

Periplasmic proteins were isolated according to the method published by Krehenbrick et al. [81]. Briefly, cells from 15 mL culture were resuspended in 1 mL of spheroplasting buffer (20% (*w/v*) sucrose, 50 mM Tris-HCl [pH 8.0], 1 mM EDTA, 0.1 mg/mL lysozyme) and mixed by gentle agitation for 30 min at room temperature. The spheroplasts were pelleted by centrifugation (10 min, 10,000× *g*, 4 °C) and obtained supernatant was used as periplasmic proteins fraction. For isolation of soluble and membrane proteins, bacterial pellet from 15 mL culture was resuspended in 1 mL 50 mM TRIS-HCl (pH 8.0) supplemented with protease inhibitors and lysozyme at a final concentration of 2 mg/mL. The solution was kept on ice for 30 min and then disrupted with the use of a FRENCH Pressure Cell Press (18 000 psi). The obtained lysate was clarified (4000× *g*) and then inclusion bodies were pelleted by centrifugation at 10,000× *g*. Obtained non-inclusion fraction was further centrifuged to pellet membrane fragments (total membrane fraction). Membranes were washed once with 50 mM Tris-HCl (pH 8.0), 1 M NaCl to solubilize membrane-associated proteins. Proteins were routinely analyzed by SDS-PAGE and either visualized with PageBlue Protein Staining Solution (Thermo Fisher Scientific, Waltham, MA, USA) or electroblotted onto PVDF membrane (Merck Millipore, Burlington, MA, USA). Native PssJ and His-tagged PssJ were detected in Western immunoblotting using chicken anti-PssJ (this work) and mouse anti-His6 antibodies (Roche, Basel, Switzerland), followed by goat anti-chicken AP-conjugated (Thermo Fisher Scientific, Waltham, MA, USA) or anti-chicken HRP-conjugated antibodies (Thermo Fisher Scientific, Waltham, MA, USA) and goat anti-mouse AP-conjugated antibodies (Cell Signaling Technology, Inc., Danvers, MA, USA). Anti-PssO and anti-PssP rabbit sera were prepared in previous works [41,82].

### 4.15. Bacterial Two-Hybrid System Assays

To construct plasmids encoding fusion proteins with CyaA, all ten RtTA1 GT-encoding genes were PCR amplified using appropriate primers listed in Table S5 and subcloned into the pKT25, pKNT25, pUT18C, and pUT18 vectors [54]. Production of fusion proteins, in either case, was confirmed through Western blotting with anti-CyaA antibodies (Santa Cruz Biotechnology, Inc., Dallas, TX, USA) and appropriate anti-mouse AP-conjugated antibodies (Cell Signaling Technology, Inc., Danvers, MA, USA). Plasmids were co-transformed into *E. coli* DHM1 strain. Initial screening for interaction was performed on LB agar plates containing 5-bromo-4-chloro-3-indolyl-β-D-galactoside (X-Gal; 40 μg/mL), isopropyl-β-D-galactopyranoside (IPTG; 0.5 mM), ampicillin (100 μg/mL), and kanamycin (40 μg/mL). For a quantitative measurement of interaction, β-galactosidase activity was measured in a plate format as described earlier [40].

### 4.16. Statistical Analyses

The results of plant tests, bacterial two-hybrid quantitative assays, biofilm formation, motility, and EPS production were submitted for statistical analyses, which were performed with Statistica 13 software (StatSoft Polska, Kraków, Poland), using one-way analysis of variance (ANOVA) and the post-hoc Tukey’s test.

## Figures and Tables

**Table 1:** Strains and plasmids used in this work.

Strain/Plasmid	Relevant Description	Reference
***Escherichia coli***		
S17-1	294 derivative RP4-2-Tc::Mu-Km::Tn7 chromosomally integrated	[52]
DH5α	*supE*44 Δ*lacU*169 (Δ80 *lacZ*ΔM15) *hsdR*17 *recA*1 *endA*1 *gyrA*96 *thi*-1 *relA*1	[53]
DHM1	Reporter strain for BTH system; F- *glnV*44(AS) *recA*1 *endA gyrA*96 *thi*-1 *hsdR*17 *spoT*1 *rfbD*1 *cya*-854	[54]
BL21(DE3)	F– *ompT hsdSB* (rB- mB-) *gal dcm* (DE3)	Novagen
***Rhizobium leguminosarum* bv. *trifolii***		
RtTA1	wild type strain, Str^R^, Rif^R^	[55]
TA1Δ*pssJ*(Gm^R^)	RtTA1 Δ*pssJ*::Gm^R^	This work
TA1Δ*pssJ*[pCM157]	RtTA1 Δ*pssJ* carrying pCM157 *cre* expressing vector	This work
TA1Δ*pssJ*	RtTA1 Δ*pssJ*	This work
TA1Δ*pssJ*[pBKpssJ-C]	Δ*pssJ* carrying pBKpssJ-C (complemented mutant)	This work
TA1Δ*pssJ*[pBKpssJ-C-His6]	ΔpssJ carrying pBKpssJ-C-His6 (complemented mutant expressing His_6_-tagged PssJ)	This work
**Plasmids**		
pET30c	*ori* pBR322, Kan^R^, *lacI,* 6xHis-tag, S-tag	Novagen
pET30c-pssJ	pET30c derivative with 827-bp fragment comprising *pssJ* gene cloned in BamHI – XhoI	This work
pCM351	*ori* ColE1, *oriT*, Ap^R^, Gm^R^, Tc^R^, allelic exchange vector	[39]
pCM157	*ori* IncP, *oriT*, Tc^R^, *cre* expression vector	[39]
pBBR1MCS-2	pBBR1 *rep*, *mob*, *lacZα* multi cloning site, Km^R^, broad-host-range cloning vector	[56]
pCGpssJ-U	pCM351 with 700 bp NdeI fragment comprising last 688 bp of *pssK* and *pssK*–*pssJ* intergenic region	This wok
pCGpssJ-UD	pCGpssJ-U with 570 bp ApaI–SacI fragment comprising *pssJ*–*pssI* intergenic region and 487 bp of *pssI*	This wok
pBKpssJ-C	pBBR1MCS-2 with 990 bp ApaI–XbaI fragment comprising last 88 bp of *pssK*, *pssK*–*pssJ* intergenic region, *pssJ*, and 68 bp downstream of *pssJ*	This work
pBKpssJ-C-His6	pBBR1MCS-2 with 940 bp ApaI–XbaI fragment comprising last 88 bp of *pssK*, *pssK*–*pssJ* intergenic region, and *pssJ* without stop codon, equipped with His6-tag coding sequence and TAA stop codon	This work
pUT18	Two-hybrid plasmid for *cyaA*T18 fusion construction, Ap^r^	[54]
pUT18C	Two-hybrid plasmid for *cyaA*T18 fusion construction, Ap^r^	[54]
pKNT25	Two-hybrid plasmid for *cyaA*T25 fusion construction, Km^r^	[54]
pKT25	Two-hybrid plasmid for *cyaA*T25 fusion construction, Km^r^	[54]
pUT18C-zip	Two-hybrid control plasmid, Ap^r^	[54]
pKT25-zip	Two-hybrid control plasmid, Km^r^	[54]
pUT18-*pssA*/*pssC*/*pssD*/*pssE/pssF*/*pssG*/*pssH*/*pssI*	pUT18 with DNA fragment of 792 bp, 981 bp, 459 bp, 477 bp, 885 bp, 975 bp, 969 bp, 945 bp comprising *pssACDEFGHI* genes cloned into XbaI-KpnI sites	This work
pUT18-*pssS*	pUT18 with DNA fragment of 1152 bp comprising *pssS* gene, cloned into XbaI-BamHI sites	This work
pUT18-*pssJ*	pUT18 with DNA fragment of 822 bp comprising *pssJ* gene, cloned into PstI-BamHI sites	This work
pUT18C-*pssA*/*pssC*/*pssD*/*pssE/pssF*/*pssG*/*pssH*/*pssI*	pUT18C with DNA fragment of 792 bp, 981 bp, 459 bp, 477 bp, 885 bp, 975 bp, 969 bp, 945 bp comprising *pssACDEFGHI* genes cloned into XbaI-KpnI sites	This work
pUT18C-*pssS*	pUT18C with DNA fragment of 1152 bp comprising *pssS* gene, cloned into XbaI-BamHI sites	This work
pUT18C-*pssJ*	pUT18C with DNA fragment of 822 bp comprising *pssJ* gene, cloned into PstI-BamHI sites	This work
pKT25-*pssA*/*pssC*/*pssD*/*pssE/pssF*/*pssG*/*pssH*/*pssI*	pKT25 with DNA fragment of 792 bp, 981 bp, 459 bp, 477 bp, 885 bp, 975 bp, 969 bp, 945 bp comprising *pssACDEFGHI* genes cloned into XbaI-KpnI sites	This work
pKT25-*pssS*	pKT25 with DNA fragment of 1152 bp comprising *pssS* gene, cloned into XbaI-BamHI sites	This work
pKT25-*pssJ*	pKT25 with DNA fragment of 822 bp comprising *pssJ* gene, cloned into PstI-BamHI sites	This work
pKNT25-*pssA*/*pssC*/*pssD*/*pssE/pssF*/*pssG*/*pssH*/*pssI*	pKNT25 with DNA fragment of 792 bp, 981 bp, 459 bp, 477 bp, 885 bp, 975 bp, 969 bp, 945 bp comprising *pssACDEFGHI* genes cloned into XbaI-KpnI sites	This work
pKNT25-*pssS*	pKT25 with DNA fragment of 1152 bp comprising *pssS* gene, cloned into XbaI-BamHI sites	This work
pKNT25-*pssJ*	pKT25 with DNA fragment of 822 bp comprising *pssJ* gene, cloned into PstI-BamHI sites	This work
pMEG65	Vector with constitutively expressed *gfp* and RK2 stabilization fragment, Tc^r^	[57]

## Supplementary Materials

Supplementary materials can be found at https://www.mdpi.com/1422-0067/21/20/7764/s1.
