# Supplementary material for: PssJ Is a Terminal Galactosyltransferase Involved in the Assembly of the Exopolysaccharide Subunit in Rhizobium leguminosarum bv. Trifolii
Source: Int J Mol Sci. 2020 Oct 20;21(20):7764. doi: 10.3390/ijms21207764 (PMC7589315; doi:10.3390/ijms21207764)
Supplement: Supplementary file 1 [file ijms-21-07764-s001.pdf]

PssJ is a terminal galactosyltransferase involved in the assembly of the exopolysaccharide subunit in *Rhizobium leguminosarum* bv. *trifolii*

Małgorzata Marczak[1*], Magdalena Wójcik[1], Kamil Żebracki[1**], Anna Turska-Szewczuk[1], Kamila Talarek[1], Dominika Nowak[1], Leszek Wawiórka[2], Marcin Sieńczyk[3], Agnieszka Łupicka-Słowik[3], Kamila Bobrek[4], Marceli Romańczuk[1], Piotr Koper[1] and Andrzej Mazur[1]**

[1] Department of Genetics and Microbiology, Institute of Biological Sciences, Maria Curie-Skłodowska University

[2] Department of Molecular Biology, Institute of Biological Sciences, Maria Curie-Skłodowska University

[3] Department of Organic and Medical Chemistry, Faculty of Chemistry, Wrocław University of Science and Technology

[4] Department of Epizootiology and Clinic of Bird and Exotic Animals, Faculty of Veterinary Medicine, Wroclaw University of Environmental and Life Sciences

[*] corresponding author

[**] contributed equally to this work

Supplementary files

Table S1. PssJ homologs revealed by ProtBLAST/PSI-BLAST similarity searches. Selected top records not described as "hypothetical" were summarized and chosen for the alignment shown in Figure 2.

**Accession number**	**Subject amino acid range**	**Predicted function**	**Organism or group of organisms (in case of metagenomic data)**	**Percentage of identity/coverage between query and subject**
RYH00067.1	3-266	galactosyl transferase	Alphaproteobacteria	50/97
TAN00599.1	1-205	galactosyl transferase, partial	Rhizobiaceae	65/75
PZU46882.1	4-264	galactosyl transferase	*Sphingomonas* sp.	50/96
TAA47749.1	22-268	galactosyl transferase	*Corallincola spongiicola*	46/91
WP_143103296.1	4-266	galactosyl transferase	*Albimonas pacifica* SFI10148.1	47/97
TIY02532.1	126-268	galactosyl transferase, partial	*Mesorhizobium* sp.	72/53
WP_158720787.1	3-244	galactosyl transferase	*Xenophilus* sp. L33	47/89
WP_134680296.1	4-264	glycosyltransferase family 2 protein	*Paracoccus* sp. YJ057	44/94
WP_155999281.1	1-266	galactosyl transferase	*Thioalkalivibrio* sp. ALJ16	44 /96

Table S2. Top ten top templates used to model PssJ structure with Phyre2.

PDB number	% coverage of query	Confidence = probability that sequences are homologous	Template information
d1xhba2	91	100	Superfamily: Nucleotide-diphospho-sugar transferases; Family: polypeptide N-acetylgalactosaminyltransferase 1, N-terminal domain
c2z86D	84	100	Molecule: chondroitin synthase; PDB title: crystal structure of chondroitin polymerase from *Escherichia coli* complexed with UDP-GlcUA and UDP
c6h4mA	95	100	Molecule: probable ss-1,3-N-acetylglucosaminyltransferase
c5tz8C	95	100	Molecule: glycosyltransferase; PDB title: crystal structure of *S. aureus* TarS
c6e4rB	89	100	Molecule: polypeptide N-acetylgalactosaminyltransferase 9; PDB title: crystal structure of the *Drosophila melanogaster* polypeptide N-2 acetylgalactosaminyltransferase PGANT9B
c2ffuA	86	100	Molecule: polypeptide N-acetylgalactosaminyltransferase 2; PDB title: crystal structure of human ppGalNAcT-2 complexed with UDP and EA2
c5nqaA	89	100	Molecule: polypeptide N-acetylgalactosaminyltransferase 4; PDB title: crystal structure of GalNAc-T4 in complex with the monoglycopeptide 3
c6pxuA	90	100	Molecule: polypeptide N-acetylgalactosaminyltransferase 12; PDB title: crystal structure of human GalNAc-T12 bound to a diglycosylated peptide, Mn2+, and UDP
c1xhbA	89	100	Molecule: polypeptide N-acetylgalactosaminyltransferase 1; PDB title: the crystal structure of UDP-GalNAc:polypeptide N-acetylgalactosaminyltransferase T1
c2d7iA	78	100	Molecule: polypeptide N- acetylgalactosaminyltransferase 10; PDB title: crystal structure of pp-GalNAc-T10 with UDP, GalNAc and Mn2+

Table S3. Results of sensitivity test of RtTA1, Δ*pssJ* and Δ*pssJ*(*pssJ*) towards ethanol (1-6%), pH of the medium (5.3-7.2) and sodium dodecyl sulfate concentration (0.01-0.05%).

			WT	Δ*pssJ*	Δ*pssJ*(*pssJ*)
Sensitivity towards	Ethanol	1%			
		2%			
		3%			
		4%			
		5%			
		6%			
	pH	5.3			
		5.7			
		6.2			
		6.7			
		7.2			
	SDS	0.01%			
		0.02%			
		0.03%			
		0.04%			
		0.05%			

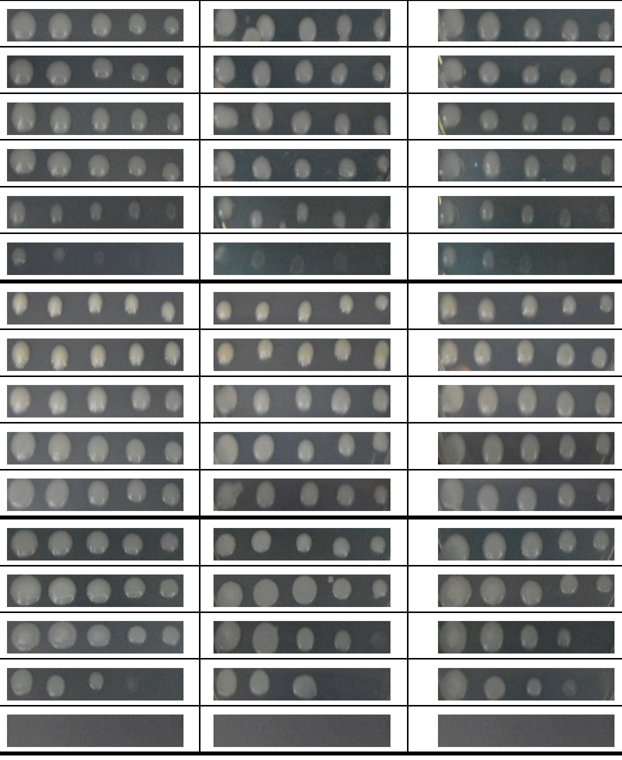

Table S4. Activity of β-galactosidase in *E. coli* DHM1 carrying two plasmids encoding bait and prey Pss proteins, either glycosyltransferases (PssA, PssC, PssD, PssE, PssS, PssF, PssG, PssH, PssI, and PssJ) or translocation/polymerization proteins PssT, PssP, PssL, and PssP2. Presented values are the means of three independent experiments with two technical repeats each. Values marked in blue represent 2-fold increase in activity relative mean value of negative controls (94.8 ± 5.1 Miller units), which is considered a positive interaction. Mean activity of β-galactosidase in positive control, i.e. for pUT18C-zip + pKT25-zip pair was 727.1 ± 43.0 Miller units.

	T25-PssA	PssA-T25
PssJ-T18	112.2 ± 26.9	100.6 ± 1.3
T18-PssJ	**865.5 ± 56.3**	100.3 ± 2.9
T25-PssJ	101.3 ± 12.2	**197.6 ± 24.4**
PssJ-T25	103.9 ± 7.2	139.0 ± 13.0
	PssA-T18	T18-PssA

	T25-PssC	PssC-T25
PssJ-T18	**187.0 ± 18.2**	**238.7 ± 25.4**
T18-PssJ	**967.8 ± 69.6**	117.7 ± 12.5
T25-PssJ	**851.2 ± 98.9**	**293.6 ±28.0**
PssJ-T25	**356.9 ±67.2**	103.2 ± 6.4
	PssC-T18	T18-PssC

	T25-PssD	PssD-T25
PssJ-T18	131.2 ± 31.2	104.8 ± 13.0
T18-PssJ	**219.0 ± 15.0**	120.0 ± 22.4
T25-PssJ	165.5 ± 15.3	123.9 ± 9.0
PssJ-T25	175.1 ± 11.2	112.3 ± 7.8
	PssD-T18	T18-PssD

	T25-PssE	PssE-T25
PssJ-T18	109.0 ± 4.0	111.7 ± 13.4
T18-PssJ	94.0 ± 10.0	121.6 ± 4.0
T25-PssJ	94.4 ± 2.2	103.5 ±2.5
PssJ-T25	106.3 ± 13.4	101.8 ± 9.8
	PssE-T18	T18-PssE

	T25-PssS	PssS-T25
PssJ-T18	101.5 ± 2.8	96.8 ± 3.3
T18-PssJ	102.9 ± 3.0	88.8 ± 17.2
T25-PssJ	105.9 ± 3.8	97.7 ± 7.0
PssJ-T25	97.8 ± 8.7	104.8 ± 7.6
	PssS-T18	T18-PssS

	T25-PssF	PssF-T25
PssJ-T18	102.4 ± 10.8	107.1 ± 10.9
T18-PssJ	**428.0 ± 10.9**	**272.3 ± 53.7**
T25-PssJ	104.4 ± 10.6	**398.4 ± 77.7**
PssJ-T25	101.9 ± 7.3	106.3 ± 9.2
	PssF-T18	T18-PssF

	T25-PssG	PssG-T25
PssJ-T18	83.6 ± 11.0	107.4 ± 5.9
T18-PssJ	**732.7 ± 42.7**	100.2 ± 7.1
T25-PssJ	180.3 ± 12.1	107.8 ± 5.8
PssJ-T25	150.9 ± 18.9	105.7 ± 9.9
	PssG-T18	T18-PssG

	T25-PssH	PssH-T25
PssJ-T18	100.1 ± 4.3	97.3 ± 6.3
T18-PssJ	**230.9 ± 29.4**	125.5 ± 14.7
T25-PssJ	132.2 ± 12.1	160.0 ± 33.1
PssJ-T25	108.4 ± 8.4	100.8 ± 6.7
	PssH-T18	T18-PssH

	T25-PssI	PssI-T25
PssJ-T18	130.2 ± 11.2	**296.3 ± 38.6**
T18-PssJ	**503.5 ± 43.0**	91.6 ± 3.5
T25-PssJ	**440.5 ± 51.1**	**312.1 ± 26.3**
PssJ-T25	**384.0 ± 93.3**	**211.7 ± 105.9**
	PssI-T18	T18-PssI

	T25-PssJ	PssJ-T25
PssJ-T18	132.9 ± 4.0	133.6 ± 14.0
T18-PssJ	**317.3 ± 56.6**	**326.3 ± 78.6**

	T25-PssP
PssJ-T18	95.7 ± 4.8
T18-PssJ	93.4 ± 2.3
T25-PssJ	104.6 ± 5.7
PssJ-T25	98.1 ± 6.7
	T18-PssP

	T25-PssT	
PssJ-T18	**216.1 ± 16.5**	
T18-PssJ	129.9 ±26.1	
T25-PssJ	104.2 ± 1.2	114.9 ±15.8
PssJ-T25	98.3 ± 3.4	106.1 ± 7.4
	PssT-T18	T18-PssT

	T25-PssL	
PssJ-T18	100 ± 1.3	
T18-PssJ	100.6 ± 4.4	
T25-PssJ	101.7 ± 9.1	100.4 ±1.5
PssJ-T25	107.9 ± 7.7	122.1 ± 0.7
	PssL-T18	T18-PssL

	T25-PssP2	
PssJ-T18	154.6 ± 9.3	
T18-PssJ	98.0 ± 2.2	
T25-PssJ	110.4 ± 4.0	112.3 ± 11.9

PssJ-T25	100.4 ± 3.7	193.9 ± 22.0
	PssP2-T18	T18-PssP2

Table S5. List of primers used in this work

Name	Sequence (5'–3')	Tm (°C)	Application
pssJ-U_FwNde	aaacatatgGCAGATCATCCAGTTCCCGCAGTC	65	amplification of genomic fragments for Δ*pssJ* mutant construction
pssJ-U_RvNde	aaacatatgCGAATGACCCCCTTAAGCCCGCAA	67	
pssJ-D_FwApa	aagggcccGCGCCGATCCCATTCGAACA	64	
pssJ-D_RvSac	agagctcCCCAGACTTTCGTCGGGTCACACG	67	
pssJ-C_FwApa	aagggcccCTCGCTCGAGGACGGAATAGA	61	amplification of genomic fragments for Δ*pssJ* mutant complementation
pssJ-C_RvXba	aatctagaTTGGTGAAGTCGAAAGAGAAAAGC	58	
pssJ-C-His6_FwApa	aagggcccCTCGCTCGAGGACGGAATAGAGTGG	66	
pssJ-C-His6_RvXba	aatctagattaatgatgatgatgatggtgCGCGGGGGTCGACCGCGTCT	72	
pCMFw1	GGGTTCCGCGCACATTTC	61	validation of cloning and sequencing of the pCM351 derivatives
pCMRv1	GCTGCGTTCGGTCAAGGT	62	
pCMFw2	CCTAACAATTCGTTCAAGCCGA	58	
pCMRv2	CGCGCGAACGACATGGAG	63	
M13pUCf	CCCAGTCACGAAGTTGTAAAACG	59	validation of cloning and sequencing of the pBBR1-MCS2 derivatives
M13pUCr	AGCGGATAACAATTTCACACAGG	58	
pUT18CFwSeq	CGGCGTGGCGGGGAAAAG	67	Sequencing of BTH plasmids derivatives
pUT18RvSeq	CGTGCGCCCGCCTGTTCA	69	
pKT25FwSeq	CAAGGGCGGCGACGATTTC	63	
pKNT25RvSeq	CCACCCCTTCGGCAATCA	61	
pssAFwBTH	AAATCTAGAAGTGACAGGGTTAACCATTGA	56	BTH cloning of *pssA* gene
pssARvBTH	AAAGGTACCCCGAAGCCTTTACCACCGGTCA	63	
pssCFwBTH	AAATCTAGAAAATCAGCAACAGACTTTTCC	53	BTH cloning of *pssC* gene
pssCRvBTH	AAAGGTACCCCGTGGGCGGCATTGGGTTTGT	69	
pssDFwBTH	AAATCTAGAAGCTGAGAAAAAATTGAAGGT	52	BTH cloning of *pssD* gene
pssDRvBTH	AAAGGTACCCCAAGGACAGCTCCTGCGTAGT	65	
pssEFwBTH	AAATCTAGAAATTCTCGTCACCGTCGGAAC	60	BTH cloning of *pssE* gene
pssERvBTH	AAAGGTACCCCGACGGCGGCAATATAATTTT	59	
pssFFwBTH	AAATCTAGAATTGAAATTATCGGTGCTTAT	49	BTH cloning of *pssF* gene
pssFRvBTH	AAAGGTACCCCTGACTGTCCTCTCCGCAGCA	67	

pssGFwBTH	AAATCTAGAAACGGATCCGAGAATTAGTGT	56	BTH cloning of *pssG* gene
pssGRvBTH	AAAGGTACCCCATGCACGACCTCCTGCGCTA	68	
pssHFwBTH	AAATCTAGAAAGCAAAGTCAAGGTTACAAT	52	BTH cloning of *pssH* gene
pssHRVBTH	AAAGGTACCCCTTTGGCGCCGACCTGAGAGT	68	
pssIFwBTH	AAATCTAGAATCGGATCTCTTCGTCAGCGT	56	BTH cloning of *pssI* gene
pssIRVBTH	AAAGGTACCCCCTGCGTCATCGTCTGAGAAA	62	
pssJ-BTH_FwPst	AAACTGCAGAACACTTGTCACCTTCATTAT	51	BTH cloning of *pssJ* gene
pssJ-pKT25_FwPst	AAACTGCAGAAACACTTGTCACCTTCATTAT	51	
pssJRvBTH	AAAGGATCCCCCGCGGGGGTCGACCGCGTCT	72	
pssSFwBTH	AAATCTAGAAAAAAAAGCCGTTATTTATGT	47	BTH cloning of *pssS* gene
pssSRvBTH	AAAGGATCCCCAGTCCGACCCCGGCTGGAAA	70	
pssJpET30Fw	AAAGGATCCTGACACTTGTCACCTTCATTATCC	57	Cloning of *pssJ* gene in pET30c vector
pssJpET30Rv	AAACTCGAGTTACGCGGGGGTCGAC	61	

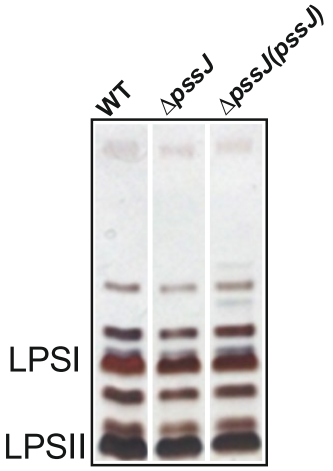

Figure S1. Lipopolysaccharide profiles of RtTA1 strain and its derivatives: Δ*pssJ* and Δ*pssJ*(*pssJ*) separated by SDS-PAGE and visualized by silver staining after oxidation with periodate. LPSI, high-molecular-weight LPS with O-antigen, LPSII, low-molecular-weight LPS, representing the lipid A-core oligosaccharide species.

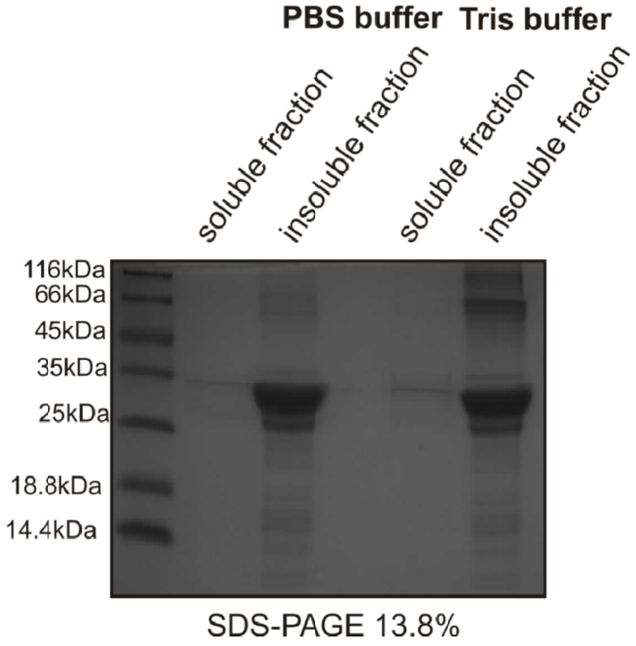

Figure S2. Refolding trial of His6-PssJ from solubilized inclusion bodies. Protein eluted form the Ni-NTA resin was subjected to refolding through overnight dialysis. No refolding was observed and 100% of protein was precipitated from the solution (insoluble fraction).

A

B

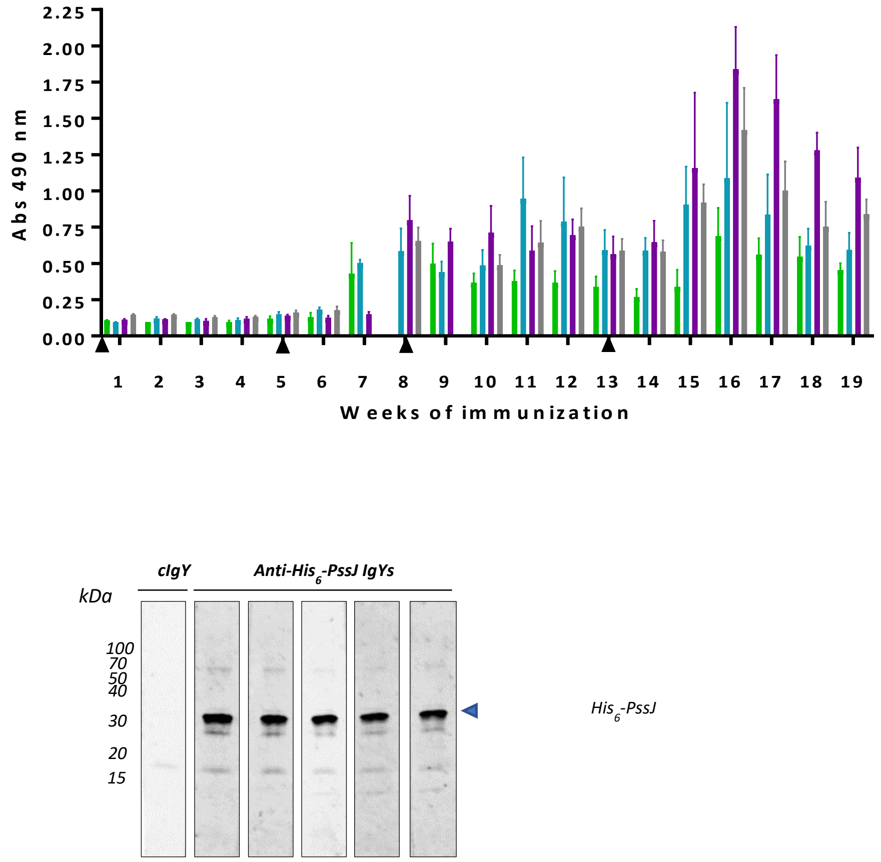

Figure S3. Development of antigen-specific IgY antibodies. (**A**) Analysis of the production of specific IgY antibodies isolated from egg yolks performed for the screening purposes (isolates from individual hens are marked with colors). Weeks of immunization are marked with arrowheads. The plate was coated with His6-PssJ protein (0.5 μg/ml) and free binding sites were blocked with 5% skimmed milk in PBST. Subsequently, wells were incubated with IgYs diluted in 0.5% skimmed milk in PBST (1:100). Detection of the resulting complexes was performed using rabbit anti-IgY IgG-HRP antibodies (1:5000) with *o*-phenylenediamine as a substrate. Results from different plates are expressed as absorbance values (Abs 490). The assigned points for specific weeks represent the mean absorbance of the measurements performed in duplicate for the eggs collected from hen in particular week. (**B**) Western blot analysis of the anti-His6-PssJ IgYs was performed after electrophoretic separation of His6-PssJ protein (100 ng/well, SDS-PAGE 4-12%, reducing conditions) and electrotransfer to a nitrocellulose membrane. After blocking the membrane, the strips were incubated with anti-His6-PssJ IgY antibodies or with control antibodies (cIgY; isolated from eggs collected from chickens after injection only with Freund`s adjuvant) diluted 100-times in 0.5% skimmed milk in PBST. Rabbit anti-IgY IgG-HRP antibodies (1:5000) were used for detection. The images were visualized with a chemiluminescent substrate and a molecular imaging system equipped with a CCD camera. The blue arrowhead indicates bands from the His6-PssJ protein.

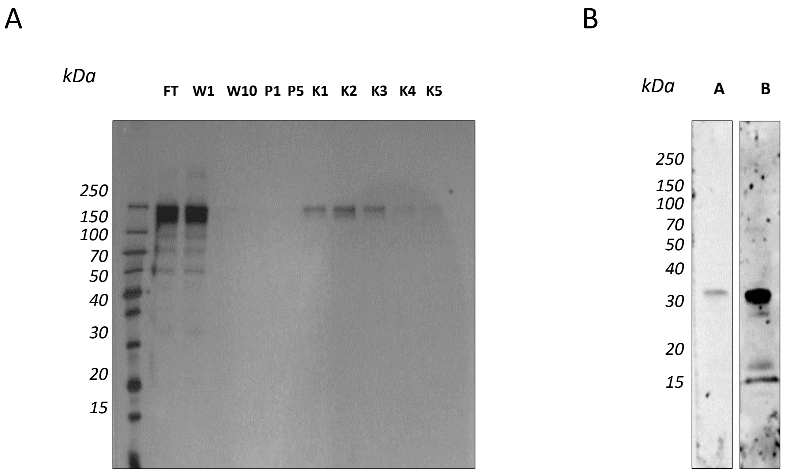

Figure S4. His_6-PssJ protein-highly reactive IgYs were purified *via* affinity chromatography in order to enrich the antibody fraction with antigen-specific immunoglobulins. For this purpose, cyanogen bromide activated resin (Thermo Scientific, Gdańsk, Poland) was modified with His_6-PssJ protein based on the manufacturer protocol. Firstly, the resin (500 mg) was preactivated with 1 mM HCl and washed with an affinity chromatography coupling buffer (100 mM sodium hydrogen carbonate, 500 mM sodium chloride, pH 8.0). Then, the solution of His_6-PssJ protein in an affinity chromatography coupling buffer and 5% DMSO was added to the resin (500 μg, 1 ml) and incubated at room temperature (2 h) and then at 4°C (overnight). Subsequently, the resin was incubated with Tris buffer for 2 h at room temperature in order to block the reactive groups of the resin and then washed alternately with Tris and acetate buffers. Affinity column was stored in PBS buffer at 4°C. In order to purify antibodies. The crude isolate of anti- His_6-PssJ IgY antibodies (150 μl) was diluted with PBS (1:1, *v/v*) and incubated with the resin for 1 h at room temperature. Unbound antibodies were removed by gravity flow (**A**, FT) and the resin was extensively washed with PBS-T (**A**, W1-W10), and PBS (**A**, P1-P5). Specific anti- His_6-PssJ IgY antibodies were eluted from the resin with citrate buffer and immediately neutralized with 1M Tris-base (**A**, K1-K5). The column was used repeatedly, each time thoroughly rinsed with PBS and stored at 4°C. K1-K5 fractions were pooled and concentrated with the use of centrifugal concentrators and their reactivity was compared in the standard Western blot (**B**) to the reactivity of the crude specific IgYs isolate (1 μg/ml in 0.5% skimmed milk in PBST) with the use of rabbit anti-IgY IgG-HRP antibodies.
